# Molecular Engineering of a Tumor‐Targeting Thione‐Derived Diketopyrrolopyrrole Photosensitizer to Attain NIR Excitation Over 850 nm for Efficient Dual Phototherapy

**DOI:** 10.1002/advs.202407727

**Published:** 2024-10-16

**Authors:** Gang Xu, Yunxia Song, Haifeng Jin, Pengmin Shi, Yubo Jiao, Fangzhou Cao, Jie Pang, Yanyan Sun, Lei Fang, Xing‐Hua Xia, Jian Zhao

**Affiliations:** ^1^ Jiangsu Province Hi‐Tech Key Laboratory for Biomedical Research School of Chemistry and Chemical Engineering Southeast University Nanjing 211189 China; ^2^ State Key Lab of Analytical Chemistry for Life Science School of Chemistry and Chemical Engineering Nanjing University Nanjing 210023 China; ^3^ School of Chemistry and Life Sciences Suzhou University of Science and Technology Suzhou 215009 China

**Keywords:** diketopyrrolopyrrole, NIR excitations, photodynamic therapy, photothermal therapy, thiocarbonyl‐substitutes

## Abstract

Photosensitizers with near‐infrared (NIR) excitation, especially above 800 nm which is highly desired for phototherapy, remain rare due to the fast nonradiative relaxation process induced by exciton‐vibration coupling. Here, a diketopyrrolopyrrole‐derived photosensitizer (DTPA‐S) is developed via thionation of carbonyl groups within the diketopyrrolopyrrole skeleton, which results in a large bathochromic shift of 81 nm, endowing the photosensitizer with strong NIR absorption at 712 nm. DTPA‐S is then introduced with a functional biomolecule (N_3_‐PEG_2000_‐RGD) via click reaction for the construction of integrin αvβ3 receptor‐targeted nano‐micelles (NanoDTPA‐S/RGD), which endows the photosensitizer with a further superlarge absorption redshift of 138 nm, thus extending the absorption maxima to ≈850 nm. Remarkably, thiocarbonyl substitution increases the nonbonding characters in frontier molecular orbitals, which can effectively suppress the nonradiative vibrational relaxation process via reducing the reorganization energy, enabling efficient reactive oxygen species (ROS) generation under 880 nm excitation. Screened by in vitro and in vivo assays, NanoDTPA‐S/RGD with high water solubility, excellent tumor‐targeting ability, and photodynamic/photothermal therapy synergistic effect exhibits satisfactory phototherapeutic performance. Overall, this study demonstrates a new design of efficient NIR‐triggered diketopyrrolopyrrole photosensitizer with facile installation of functional biomolecules for potential clinical applications.

## Graphical abstract

This study presents a new design of diketopyrrolopyrrole (DPP) photosensitizer via thiocarbonyl substitution, which not only improves the photo‐physicochemical properties of the photosensitizer but also provides a novel strategy to overcome the energy gap law. The prepared DPP scaffold is a promising platform for the construction of efficient NIR‐excited photosensitizers for potential clinical applications.

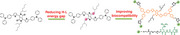

## 1 Introduction

Narrow band gap photosensitizers with strong absorption in near‐infrared regions (NIR, > 700 nm) have gained great interest for their deep tissue penetration and less photodamage in photodynamic therapy (PDT).^[^ ^1^, ^2^, ^3^, ^4^ ^]^ However, only a few NIR dyes hold potential for clinical use as photosensitizers in PDT due to the innate limitation known as “energy gap law” which indicates that the nonradiative rate (*k*_nr_) increases exponentially with decreasing the energy gap.^[^ ^5^, ^6^, ^7^ ^]^ Therefore, the nonradiative decay originating from vibrational relaxation becomes the main deactivation process for NIR dyes, eventually resulting in poor or negligible reactive oxygen species (ROS) generation.^[^ ^8^ ^]^ In contrast, the fast nonradiative decay of NIR dyes is favorable for photothermal therapy (PTT) with efficient heat generation.^[^ ^9^ ^]^ Of note, single PDT or PTT has inherent deficiencies in fighting against the tumor microenvironment, and the superior therapeutic efficacy of combination therapy of PDT and PTT has been extensively validated.^[^ ^10^, ^11^, ^12^ ^]^ As a result, developing novel NIR‐absorbing photosensitizers with potent ROS generation to achieve a synergistic effect with PTT is a promising way to overcome the limitations of PDT.

Among the existing NIR dyes, most are derived from the structures of boron‐azadipyrromethene (aza‐BODIPY) and cyanine,^[^ ^13^, ^14^ ^]^ which often suffer from serious photobleaching and inefficient intersystem crossing (ISC) transitions. Diketopyrrolopyrrole (DPP) is an excellent fluorescent dye with inherent advantages of high photostability, large extinction coefficient, and intensive fluorescence emission.^[^ ^15^, ^16^, ^17^, ^18^, ^19^, ^20^ ^]^ The high planarity and electron deficiency make DPP serve as an efficient electron acceptor, thus favoring less oxygen‐dependent type‐I PDT reactions via electron transfer processes, which can overcome the hypoxia microenvironment of tumor tissue to some extent.^[^ ^21^, ^22^, ^23^ ^]^ To date, it is still a great challenge to develop DPP‐based NIR‐absorbing dyes because the characteristic structured absorption band of the DPP core is mainly located at 400–550 nm, which is far from the NIR region. One effective strategy to red‐shift the absorption bands of DPPs is to introduce the π‐conjugated dyes or building blocks such as porphyrin, boron dipyrromethane, and benzodithiophene.^[^ ^24^, ^25^, ^26^, ^27^, ^28^ ^]^ However, ROS generation efficiency is low or negligible for DPP‐based dyes with the absorption in the deep‐red and NIR regions due to the “energy gap law”.^[^ ^29^, ^30^, ^31^, ^32^ ^]^ In addition, DPP‐derived photosensitizers are hydrophobic in nature with poor aqueous solubility and tumor‐targeting ability due to their large and/or lengthy π‐conjugated structures, which undoubtedly limits the therapeutic efficacy and further application. Therefore, a feasible and effective strategy is needed to improve the photo‐physicochemical properties of DPP‐based photosensitizers as well as increase their aqueous solubility and tumor‐targeting abilities.

Thiobases are a class of DNA or RNA nucleobases with sulfur‐for‐oxygen replacement, which have shown attractive phototherapeutic effects and unique biological functions, and thus have been widely explored as photo‐chemotherapeutic agents for cancer treatment.^[^ ^33^, ^34^ ^]^ Inspired by the thiobases, we propose that the thionation of carbonyl groups in DPP has the potential to improve its photo‐physicochemical properties with the following advantages.^[^ ^35^, ^36^, ^37^, ^38^ ^]^ First, the absorption maxima of DPP‐based dyes could be red‐shifted via thiocarbonyl substitution due to the lowering of the energy of the lowest unoccupied molecular orbital (LUMO).^[^ ^39^, ^40^, ^41^ ^]^ Second, the stronger spin‐orbit coupling constant (SOC values: ζ_S_ = 397 cm^−1^ *vs*. ζ_O_ = 152 cm^−1^) of sulfur relative to oxygen has the potential to improve the ISC efficiencies of the thiocarbonyl‐substituted DPP derivatives.^[^ ^42^, ^43^ ^]^

**Scheme 1:**
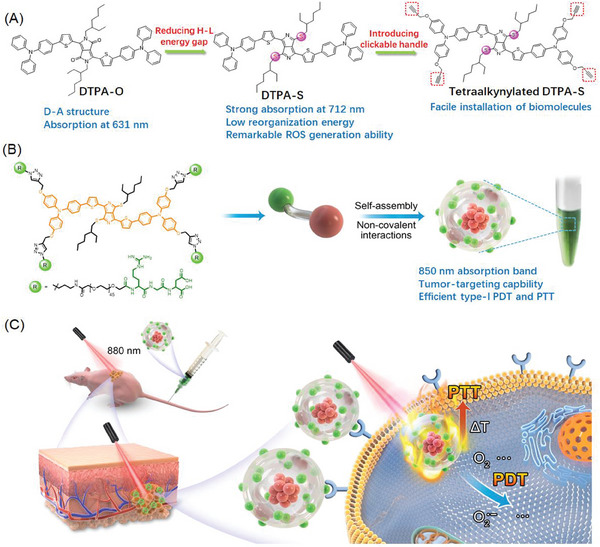
Structure of DTPA‐S and schematic illustration of NanoDTPA‐S/RGD for efficient tumor targeting and type‐I PDT/PTT synergistic therapy. A) The design strategy of DTPA‐S and alkynylated DTPA‐S based on the DTPA‐O molecule. B) Chemical structure, aggregated and dispersed states of NanoDTPA‐S/RGD. C) The working mechanism of NanoDTPA‐S in tumor targeting and dual phototherapy.

We herein developed a thiocarbonyl‐substituted DPP photosensitizer (DTPA‐S, **Scheme** **1**A) with strong NIR absorption at 712 nm, which shows a large bathochromic shift (81 nm) compared with the unsubstituted compound (DTPA‐O) because of the stabilization of the LUMO energy level. Moreover, thiocarbonyl substitution could effectively suppress the nonradiative decay process due to the increased nonbonding characters in LUMO,^[^ ^44^, ^45^ ^]^ thus enabling efficient ROS generation under NIR excitation.^[^ ^8^ ^]^ Consequently, thiocarbonyl substitution not only red‐shifts the absorption band of the photosensitizer but also provides a novel strategy to overcome the energy gap law, which is essential for the development of high‐efficiency and long‐lived NIR photosensitizers.

To address the limitation of poor aqueous solubility and tumor targeting capability, a novel NIR scaffold (tetraalkynylated DTPA‐S) with four terminal alkynes as clickable handles was developed for facile installation of functional biomolecules. Based on the prepared NIR scaffold, N_3_‐PEG_2000_‐RGD was introduced for the construction of integrin α_v_β_3_ receptor‐targeted nano‐micelles (NanoDTPA‐S/RGD, Scheme 1B).^[^ ^46^ ^]^ The self‐assembly of the micelles further significantly extends the absorption maxima by 138 nm to ≈850 nm. NanoDTPA‐S/RGD with potent type‐I ROS generation, efficient photothermal performance, and high aqueous solubility and tumor‐targeting ability exhibit a significant tumor suppressive effect both in vitro and in vivo under 880 nm excitation (Scheme 1C). Up till now, only a few examples of DPP‐based photosensitizers with excitation beyond 700 nm have been reported,^[^ ^47^ ^]^ let alone above 800 nm. Therefore, the designed thiocarbonyl‐substituted DPP‐based scaffold with facile functionalization features is a promising platform for the construction of highly efficient NIR‐excited photosensitizers.

## 2 Results and Discussion

### 2.1 Design and Synthesis of DTPA‐S

**Figure 1:**
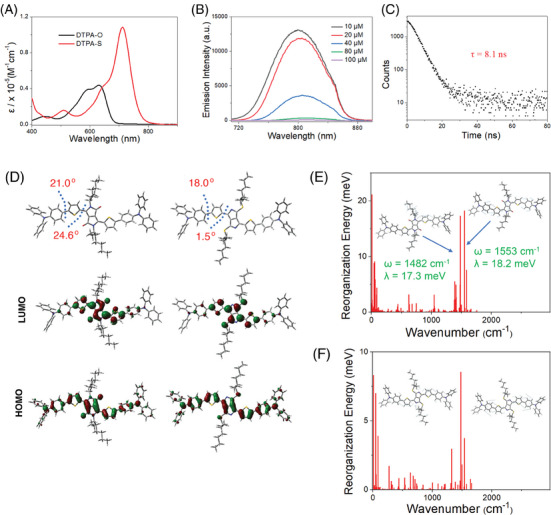
Photophysical behaviors and quantum‐chemical calculations of DTPA‐S. A) Absorption spectra of DTPA‐O and DTPA‐S in dichloromethane. B) Emission spectra of DTPA‐S at various concentrations (λ_ex_ = 650 nm). C) Emission decay curves of DTPA‐S in dichloromethane. D) Optimized ground‐state (S_0_) geometries, and calculated HOMOs, LUMOs, and energy levels of DTPA‐O and DTPA‐S. Mode‐specific reorganization energies of E) DTPA‐O and F) DTPA‐S between S_1_ and S_0_ states.

DTPA‐S was prepared through a four‐step reaction starting from 3,6‐di(2‐thienyl)‐2,5‐dihydropyrrolo[3,4‐c]pyrrole‐1,4‐dione (Scheme S1, Supporting Information). Besides, the unsubstituted carbonyl compound (DTPA‐O) was synthesized as the control compound.^[^ ^48^, ^49^ ^]^ The chemical structure of DTPA‐S was confirmed by ^1^H and ^13^C NMR spectroscopy, and MALDI‐TOF‐MS spectrometry (Figures S1–S3, Supporting Information). The purity of DTPA‐S was determined by High‐performance liquid chromatography with a purity of 97.88% (Figure S4, Supporting Information). The absorption spectra of DTPA‐S in dichloromethane (**Figure** **1**A) show an intense NIR absorption band at 712 nm with a molar extinction coefficient of 1.08×10^5^ m ^−1^ cm^−1^, indicating its excellent light‐harvesting ability in the NIR region. The thiocarbonyl substitution results in a larger bathochromic shift and enhances the molar extinction coefficient, which is highly desired for phototherapy.

### 2.2 Optical Properties

The emission peak of DTPA‐S appears at 799 nm in dichloromethane (Figure 1B), which is slightly red‐shifted with the increase of DTPA‐S concentrations, attributed to the aggregation. Besides, the aggregation decreases the fluorescence intensity of DTPA‐S, which is favorable for maximizing the multimodal synergistic phototherapy via suppression of the radiative transition. The radiative quantum yield of DTPA‐S is 0.9% at 10 µm, which is much lower than that of DTPA‐O (7.9%). The emission lifetime is 8.1 ns for DTPA‐S (Figure 1C), which is longer than that of DTPA‐O (3.9 ns, Figure S5, Supporting Information). Then, *k*_nr_ can be calculated based on the radiative quantum yield and emission lifetime, which are 1.22 ×10^8^ s^−1^ and 2.36×10^8^ s^−1^ for DTPA‐S and DTPA‐O.

Significantly, thiocarbonyl substitution effectively suppresses the nonradiative deactivation process of DTPA‐S, despite having a narrow band gap, which is contrary to the energy gap law. The photostability of DTPA‐S was evaluated by absorption spectra together with an FDA‐approved NIR dye (indocyanine green, ICG) for comparison. As shown in Figure S6 (Supporting Information), the absorption intensity of ICG decreases fast upon irradiation, whereas negligible changes are observed for DTPA‐S, indicating the high photostability of DTPA‐S.

### 2.3 Theoretical Calculation

To explore the underlying mechanism of decreased *k*_nr_, a theoretical calculation was performed to investigate the electronic natures and bonding characteristics of DTPA‐S and DTPA‐O. As presented in Figure 1D, thiocarbonyl substitution has a profound effect on the spatial structure of DTPA‐S, which reduces the dihedral angle between DPP and thiophene from 24.6 ° (DTPA‐O) to 1.5 ° (DTPA‐S), thus improving the planarity of the molecule. Besides, the energy of the LUMO in DTPA‐S is ‐2.90 eV, which is lower than that of DTPA‐O (‐2.45 eV), thus leading to a narrow energy gap between the highest occupied molecular orbital (HOMO) and LUMO.

The LUMOs of DTPA‐O and DTPA‐S are similar, and their orbitals are mainly localized on the DPP and thiophene units. A closer examination of the LUMOs reveals that DTPA‐S shows an enhanced nonbonding molecular orbital character in the DPP core compared to DTPA‐O. Notably, nonbonding could result in much fewer bond length variations after excitation, thus favoring to reduction of the molecular internal reorganization energy.^[^ ^44^, ^45^ ^]^ The reduced reorganization energy can lower the Franck‐Condon overlap between the ground and excited vibronic states, thereby effectively inhibiting the non‐radiative decay processes.

In order to further confirm our assumption, the internal reorganization energies of DTPA‐O and DTPA‐S between the lowest singlet excited state (S_1_) and ground state (S_0_) were calculated based on the normal‐mode analysis approach. The reorganization energy of DTPA‐O is 402.3 meV, which is over two‐fold higher than that of DTPA‐S (148.0 meV), suggesting that the internal geometry relaxation is greatly inhibited in DTPA‐S. As presented in Figure 1E,F, the reorganization energies of DTPA‐S for both the low‐ and high‐frequency vibrational modes are decreased as compared to DTPA‐O, suggesting the decoupling of the exciton from the vibrations of DTPA‐S. The largest reorganization energy of DTPA‐O in the high‐frequency region is 18.2 meV for a stretching vibrational mode of 1553 cm^−1^, which is about five‐fold higher than that of the similar vibrational mode (3.7 meV, 1543 cm^−1^) of DTPA‐S, indicating that thiocarbonyl substitution has a prominent suppression effect on the exciton‐vibration coupling.

### 2.4 Photo‐physicochemical Properties

**Figure 2:**
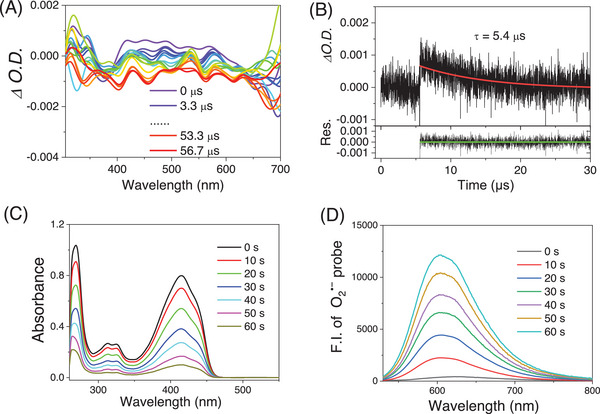
A) Nanosecond time‐resolved transient absorption spectra of DTPA‐S under 690 nm pulsed laser excitation. B) Decay trace of DTPA‐S at 470 nm in deaerated dichloromethane (5.0 × 10^−5^ m). C) Absorption spectra of DPBF in the presence of DTPA‐S under 730 nm (0.3 W cm^−2^) laser irradiation in dichloromethane. D) Time‐dependent fluorescence spectra of DHE (50 µm) in the presence of ctDNA (250 µg mL^−1^) and DTPA‐S (20 µm) encapsulated with pluronic F127 in water under 730 nm laser irradiation (0.3 W cm^−2^).

The triplet‐state behaviors of DTPA‐O and DTPA‐S were investigated by nanosecond laser flash photolysis. No transient absorption signal was detected for DTPA‐O, while DTPA‐S could undergo intersystem crossing to the triplet state with a lifetime of 5.4 µs under identical conditions, despite the triplet transient absorption signal being weak (**Figure** **2**A,B). The long‐lived triplet state of DTPA‐S confirms the suppression of the non‐radiative decay process.

It is worth noting that although a few small‐molecule DPP dyes have been ingeniously developed with NIR absorption, e.g. conjugation with porphyrin,^[^ ^30^ ^]^ no or negligible ROS generation is observed for these dyes. Thus, the ROS generation ability of DTPA‐S was evaluated under 730 nm laser irradiation. First, 1,3‐diphenyl‐isobenzofuran (DPBF) was used as a total ROS indicator. Figure 2C shows that DPBF is decomposed within one minute, indicating the efficient ROS generation capability of DTPA‐S. To further identify the ROS type, 9,10‐anthracenediylbis(methylene)‐dimalonic acid (ABDA) and dihydroethidium (DHE) were used as the trapping agents of singlet oxygen (^1^O_2_) and O_2_˙ˉ, respectively. As presented in Figure S7 (Supporting Information), undetectable changes in ABDA absorption spectra are observed under irradiation, suggesting that DTPA‐S can hardly induce the generation of ^1^O_2_. In contrast, the emission intensity of DHE increases over time upon irradiation (Figure 2D), which is indicative of the generation of O_2_˙ˉ. Similar photochemical behaviors of DTPA‐O are observed except a 635 nm laser is used as the light source (Figure S8, Supporting Information). It is demonstrated that electron‐deficient substrates are highly desired for the development of type‐I photosensitizers because electron‐deficient substrates favor electron transfer.^[^ ^50^ ^]^ Thus, the electron deficiency of DPP is beneficial for O_2_˙ˉ generation via electron transfer. Overall, the above studies indicate that DTPA‐S can produce ROS generation via a type‐I photosensitization process.

### 2.5 Design and Preparation of NanoDTPA‐S/RGD

**Figure 3:**
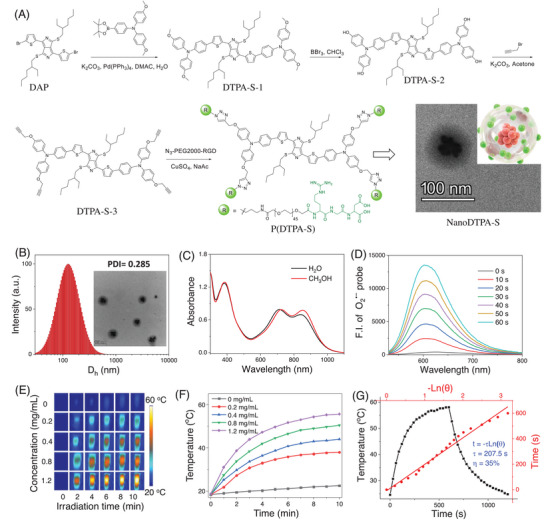
A) Synthesis route of P(DTPA‐S/RGD) and the self‐assembly of P(DTPA‐S/RGD) to form NanoDTPA‐S/RGD in aqueous solution. B) DLS profile and TEM image (inset) of NanoDTPA‐S/RGD. C) The absorption spectra of NanoDTPA‐S/RGD in methanol and water. D) The time‐dependent fluorescence spectra of DHE (50 µm) in the presence of ctDNA (250 µg mL^−1^) and NanoDTPA‐S/RGD upon 880 nm (0.3 W cm^−2^) laser irradiation. E) Time‐ and concentration‐dependent thermal images of NanoDTPA‐S/RGD under 880 nm (0.3 W cm^−2^) laser irradiation. F) Temperature curves of NanoDTPA‐S/RGD, which is the quantitative data of D. G) Photothermal conversion profile and cooling time vs −ln(θ) plot of NanoDTPA‐S/RGD.

In order to improve the in vitro and in vivo therapeutic performance of DTPA‐S, four terminal alkynes were incorporated into DTPA‐S as clickable handles via three synthetic steps (**Figure** **3**A; Figures S9–S14, Supporting Information). Then, the integrin α_v_β_3_ receptor‐targeted functional molecule N_3_‐PEG_2000_‐RGD reacted with alkynylated DTPA‐S to form an ABA‐type amphiphilic copolymer (NanoDTPA‐S/RGD) with molecular weight ranging from ca. 11 000–12 500 peaked at 11 656 (Figure S15, Supporting Information). The ^1^H NMR spectra show the characteristic chemical shifts for PEG at 3.63 ppm and RGD in the ranges of 1.2‐1.9 ppm and 2.4–3.4 ppm (Figure S16, Supporting Information). Then, gel permeation chromatography (GPC) was exploited to evaluate the purity of NanoDTPA‐S/RGD in the medium of PBS (pH = 7.4). As shown in Figure S17 (Supporting Information), only one peak was observed, indicating that NanoDTPA‐S/RGD has a good purity. The amphiphilic copolymer can self‐assemble into well‐defined core‐shell spherical micelles in aqueous solution with the solubility of 21.5 mg mL^−1^ at 25 °C, thus converting DTPA‐S from totally insoluble to well soluble in aqueous solution. The hydrodynamic diameter of NanoDTPA‐S/RGD was determined to be ca. 124 nm using dynamic light scattering (DLS, Figure 3B), which is suitable for penetration and accumulation in tumor tissues via enhanced permeability and retention (EPR) effect. The stability of NanoDTPA‐S/RGD was tested in PBS (pH = 5.4, 7.4, and 9.0), FBS, and DMEM using UV‐vis absorption spectra at different time points. As exhibited in Figure S18 (Supporting Information), negligible changes in the absorption bands of NanoDTPA‐S/RGD were observed, indicating the good stability of NanoDTPA‐S/RGD in different media and pH values.

The photo‐physicochemical properties of NanoDTPA‐S/RGD were then studied. As presented in Figure 3C, a new absorption band at ca. 850 nm appears for NanoDTPA‐S/RGD. As a result, the introduction of hydrophilic PEG into DTPA‐S is favorable for the formation of an amphiphilic block copolymer, which could facilitate the intermolecular aggregation between DTPA‐S, thus resulting in bathochromic shift of the electronic absorption band. It has been demonstrated that achieving an aggregated state with a redshift larger than 100 nm is a great challenge.^[^ ^51^ ^]^ Here, the self‐assembly endows the micelles with a superlarge absorption redshift of 138 nm, which is very impressive. No emission peak was detected at either 77 or 293 K, implying that NanoDTPA‐S/RGD can maximize the photoinduced therapeutic efficacy by suppressing the radiative transition process. Remarkably, NanoDTPA‐S/RGD can maintain the photochemical activity of DTPA‐S with efficient ROS and O_2_˙ˉ generation (Figure 3D; Figures S19 and S20, Supporting Information), and achieve deep penetration upon 880 nm laser irradiation, favoring deep‐seated or large solid tumor treatment. The ROS quantum yield of NanoDTPA‐S/RGD was calculated to be 16.2% with DPBF as a probe. Additionally, electron spin resonance (ESR) spectroscopy with 2,2,6,6‐tetramethyl‐4‐piperidone (TEMP) and 5,5‐dimethyl‐1‐pyrroline N‐oxide (DMPO) as the spin‐trapping agents were utilized to identify the ROS species generated by NanoDTPA‐S/RGD. As shown in Figure S21 (Supporting Information), no ESR signal of 2,2,6,6‐tetramethyl‐4‐piperidone‐N‐oxyl (TEMPO) generated by the reaction of TEMP with ^1^O_2_ was observed as the irradiation time increased from 0 to 2 min. However, the ESR signal of DMPO‐OOH adducts was detected, indicating the generation of O_2_^•−^. These results indicate the ability of NanoDTPA‐S/RGD to promote ROS generation via a type I photosensitization process.

### 2.6 Photothermal Property

The photothermal properties of NanoDTPA‐S/RGD were evaluated under 880 nm laser irradiation. Obviously, NanoDTPA‐S/RGD induces the heat generation in a concentration‐dependent manner (Figure 3E). A high‐temperature increase (Δ*T*) of 36.9 °C is recorded at the concentration of 1.2 mg mL^−1^ for 10 min irradiation, enabling to cause cell death via PTT (Figure 3F). The photothermal conversion efficiency (PCE) was determined to be 35.0% based on the heating/cooling curves (Figure 3G), which is higher than commonly used photothermal materials, such as gold nanorods (21.0%) and gold nanoshells (13.0%).^[^ ^52^ ^]^ Moreover, NanoDTPA‐S/RGD shows excellent photothermal stability as evidenced by no decay in the photothermal temperature plateau after five irradiation/cooling cycles, whereas ICG exhibits poor photothermal stability (Figure S22, Supporting Information).

### 2.7 In Vitro Photocytotoxicity

**Figure 4:**
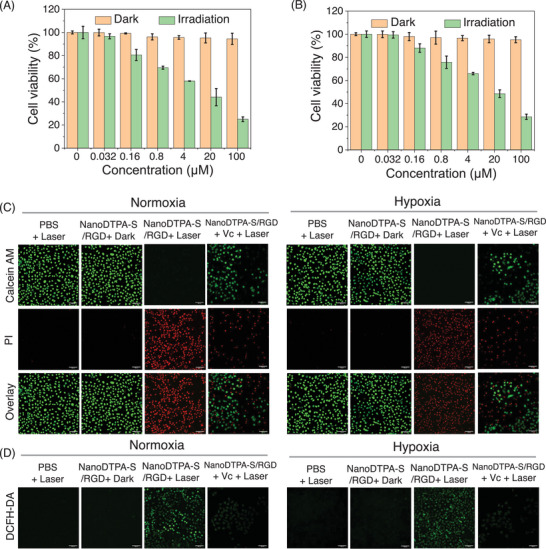
Relative cell viability of A549 cells treated with NanoDTPA‐S/RGD at various concentrations under A) normoxia and B) hypoxia in darkness or under laser irradiation. C) Confocal fluorescence images of A549 cells stained with calcein AM (green, live cells)/propidium iodide (red, dead cells) under different treatments (scale bar: 100 µm). D) Confocal fluorescence images of DCFH‐DA stained A549 cells under different experimental conditions (scale bar: 100 µm). Irradiation performed with an 880 nm laser at 0.3 W cm^−2^ for 10 min (180 J cm^−2^).

The human non‐small‐cell lung cancer A549 cells were demonstrated to have high expression of integrin α_v_β_3_ receptor.^[^ ^53^ ^]^ Thus, the in vitro photocytotoxicity of NanoDTPA‐S/RGD was investigated against A549 cells under both normoxic and hypoxic (2% O_2_) conditions using MTT ([3‐(4,5‐dimethylthiazol‐2‐yl)‐2,5‐diphenyltetrazolium bromide]) assay with ICG as positive control. As presented in **Figure** **4**A,B, NanoDTPA‐S/RGD‐treated A549 cells show normal viability in the absence of laser irradiation, suggesting the negligible dark cytotoxicity of NanoDTPA‐S/RGD. Once laser irradiation is administered, the cell viability is decreased obviously in a dose‐dependent manner, with IC_50_ values of 8.8 ± 0.7 and 15.2 ± 0.8 µm under normoxia and hypoxia, respectively, which were much lower than those of ICG (46.5 ± 0.9 and >100 µm for normoxia and hypoxia, respectively). The potent photocytotoxicity under hypoxia indicates that NanoDTPA‐S/RGD is a less oxygen‐dependent photosensitizer, which can overcome the tumor hypoxia‐associated PDT resistance to some extent. The in vitro photocytotoxicity was further investigated in the presence of ROS scavenger Vitamin C (Vc). As exhibited in Figure S23 (Supporting Information), the cell viability increases upon the addition of Vc with IC_50_ values of 26.4 ± 1.4 and 28.2 ± 2.4 µm for NanoDTPA‐S/RGD under normoxic and hypoxic conditions, respectively, indicating a photodynamic/photothermal synergistic effect of NanoDTPA‐S/RGD.

To confirm the NanoDTPA‐S/RGD‐mediated photocytotoxicity, A549 cells treated with NanoDTPA‐S/RGD were stained with calcein AM and propidium iodide (PI) to label the living and dead cells, respectively in the presence and absence of laser irradiation. As presented in Figure 4C, NanoDTPA‐S/RGD‐treated A549 cells display intense red fluorescence upon 880 nm laser irradiation under both normoxic and hypoxic conditions, demonstrating the considerable cell death caused by NanoDTPA‐S/RGD‐mediated phototherapy. On the contrary, negligible dead A549 cells are observed without laser irradiation under either normoxic or hypoxic conditions as revealed by the green fluorescence, validating the low dark cytotoxicity of NanoDTPA‐S/RGD. Additionally, both live and dead A549 cells are observed when ROS scavenger Vc is added in the NanoDTPA‐S/RGD plus laser group, indicating the death of cells is partly caused by ROS. The ROS generation efficiencies in NanoDTPA‐S/RGD‐treated A549 cells were also investigated using non‐fluorescent 2,7‐dichlorodihydrofluorescein diacetate (DCFH‐DA) as a probe, which can be oxidized into fluorescent DCF by ROS. As presented in Figure 4D, no fluorescence is observed for NanoDTPA‐S/RGD‐treated A549 cells in the dark, whereas green fluorescence is evident once irradiation was conducted, suggesting the potent ROS generation capability of NanoDTPA‐S/RGD under either normoxia or hypoxia. The ROS generation capability of NanoDTPA‐S/RGD was also measured by flow cytometry. As shown in Figure S24 (Supporting Information), the intracellular ROS levels in A549 cells under normoxia and hypoxia were significantly increased in the NanoDTPA‐S/RGD + 880 nm laser group, confirming their excellent ROS generation abilities. However, the intracellular ROS level in NanoDTPA‐S/RGD‐treated A549 cells was negligible for the control group without laser irradiation or in the presence of Vc.

### 2.8 In Vivo Antitumor Efficacy

**Figure 5:**
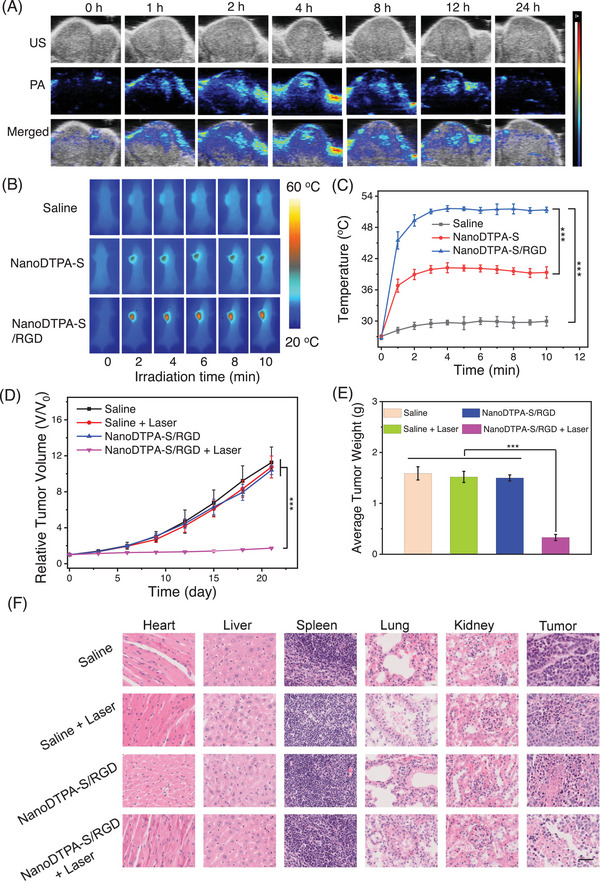
A) in vivo PA images of the NanoDTPA‐S/RGD‐treated A549 tumor‐bearing mice recorded under 880 nm illumination. B) Photothermal profiles of A549 tumor‐bearing mice treated with saline, NanoDTPA‐S, and NanoDTPA‐S/RGD upon 880 nm laser irradiation (0.3 W cm^−2^) for different times. C) Temperature elevations at the tumor sites, which is the quantitative data of (B). D) Relative tumor growth curves of A549 tumor‐bearing mice with different treatments. E) Average tumor weights of A549 tumor‐bearing mice after different treatments (***p < 0.001 determined by Student's t‐test). F) Histological analysis of the major organs and tumors after different therapeutic processes. The scale bar is 40 µm for all panels.

The in vivo behaviors of micelles were further evaluated in A549‐bearing BALB/c nude mice. The photoacoustic (PA) imaging technique was applied to illustrate the accumulation profiles of NanoDTPA‐S/RGD at the tumor site. Besides, the micelles without the RGD group were prepared as a control (denoted as NanoDTPA‐S, Scheme S2, Supporting Information), which also exhibits a well‐defined spherical morphology with a hydrodynamic diameter of ca. 104 nm (Figure S25, Supporting Information). As shown in Figure S26 (Supporting Information), both of the micelles are efficient PA imaging probes with strong PA signals under 880 laser excitation, which is increased in a concentration‐dependent manner. Therefore, the in vivo PA signals of NanoDTPA‐S/RGD and NanoDTPA‐S were monitored with 880 nm illumination at different time points after the tail vein injection of A549 tumor‐bearing mice. **Figure** **5**A shows that the PA signals of NanoDTPA‐S/RGD are progressively enhanced after injection, and achieved maximal intensity at 4 h post‐injection with a 15.2‐fold increase compared to that of 0 h. NanoDTPA‐S reaches a plateau at 8 h post‐injection with an intensity increase of 8.2‐fold, which is lower than that of NanoDTPA‐S/RGD (Figure S27, Supporting Information), suggesting the superior tumor‐targeting ability of NanoDTPA‐S/RGD. Remarkably, the PA signals of NanoDTPA‐S/RGD gradually disappear over time, implying that the prepared micelles could be easily excreted from the body.

The in vivo photothermal heating profiles of NanoDTPA‐S/RGD were investigated in A549 tumor‐bearing mice together with NanoDTPA‐S for comparison. As presented in Figure 5B,C, a small temperature elevation (Δ*T* ≈ 2.9 °C) is detectable for the saline‐treated mice, whereas the significant temperature increases are observed for NanoDTPA‐S‐ and NanoDTPA‐S/RGD‐treated mice, indicating the efficient in vivo heat‐generating capabilities of NanoDTPA‐S and NanoDTPA‐S/RGD. Remarkably, the treatment of NanoDTPA‐S/RGD results in a higher temperature elevation as compared to NanoDTPA‐S, possibly attributing to the superior tumor targeting ability of NanoDTPA‐S/RGD.

Encouraged by the excellent in vivo performance of NanoDTPA‐S/RGD, the phototherapeutic efficacy of NanoDTPA‐S/RGD‐mediated PDT/PTT was evaluated on A549 tumor‐bearing mice. Female BALB/c mice with a tumor volume of 100 mm^3^ were divided randomly into four groups (*n* = 4), including i) Saline group, ii) Saline + 880 nm laser group, iii) NanoDTPA‐S/RGD group, and iv) NanoDTPA‐S/RGD + 880 nm laser group. The mice were injected in the tail vein in 1, 7, and 14 days (2.0 mg kg^−1^), and the tumor sites were exposed to an 880 nm laser for 10 min at 4 h post‐injection. As shown in Figure 5D, the tumor volumes of groups i–iii increase remarkably during the 21‐day treatment period, indicating that neither compound NanoDTPA‐S/RGD nor laser exposure has an obvious effect on tumor growth. In sharp contrast, the tumor volume of the NanoDTPA‐S/RGD plus laser group remains stagnated with an effective tumor growth inhibition (TGI) of 79.09% (Figures S28 and S29, Supporting Information). The tumor weight data confirm the excellent anti‐tumor efficacy of NanoDTPA‐S/RGD under 880 nm laser irradiation (Figure 5E).

Hematoxylin and eosin (H&E) staining of the major organs (heart, liver, spleen, lung, and kidney) as well as tumor tissue were examined. The H&E staining images of tumors displayed in Figure 5F show that NanoDTPA‐S/RGD plus laser irradiation could cause severe cancer cell destruction, demonstrating the excellent in vivo phototherapeutic efficacy of NanoDTPA‐S/RGD. In contrast, no obvious pathological alternations or damage is observed for all organs, suggesting the high biosafety of NanoDTPA‐S/RGD. Additionally, the mice did not show any significant differences in body weights (Figure S30, Supporting Information), behaviors, and appearance. These findings collectively demonstrate the negligible in vivo toxicity of NanoDTPA‐S/RGD.

## 3 Conclusion

In summary, a thiocarbonyl‐substituted NIR‐triggered photosensitizer, DTPA‐S, was designed and synthesized, which shows attractive photo‐physicochemical properties as compared to its carbonyl analog, including red‐shifted absorption spectra, enhanced molar extinction coefficient, and reduced nonradiative rate, highlighting its application potential in phototherapy. Theoretical calculations reveal that thiocarbonyl substitution can stabilize the LUMO energy level and increase the nonbonding character of LUMO, thus resulting in reduced energy band gap and internal reorganization energy, respectively. N_3_‐PEG‐RGD was further introduced to DTPA‐S via click reaction to form a core‐shell nanostructure, which can not only improve the water solubility and tumor targeting capability of the photosensitizer but also greatly red‐shifted the absorption maxima to ≈850 nm. Moreover, NanoDTPA‐S/RGD displays highly efficient synergistic type‐I PDT/PTT therapy both in vitro and in vivo. This study provides effective evidence that the thiocarbonyl‐substituted DPP‐based skeleton is a promising platform for the construction of NIR‐triggered phototherapeutic agents in future therapeutic applications.

## 4 Experimental Section

### Materials and Instrumentation

All chemicals and solvents were purchased from commercial suppliers, and used without additional purification. mPEG_2000_‐N_3_ and N_3_‐PEG_2000_‐RGD were provided by Xi'an Qiyue Biology LTD (China). Mass spectrometry (MS) was undertaken on a Bruker UltrafleXtreme MALDI‐TOF MS instrument. ^1^H‐NMR and ^13^C‐NMR spectra were performed on a Bruker Avance III‐HD 600 MHz spectrometer using CDCl_3_ or d_6_‐DMSO as solvents at room temperature. The chemical shifts (δ) were reported in ppm (parts per million) using tetramethylsilane (TMS) as an internal standard. Elemental analysis of C, H, and N was carried out on a Vario MICROCHNOS elemental analyzer (Elementar). High‐performance liquid chromatography (HPLC) experiments were acquired on a Waters Alliance e2695 chromatograph. Gel permeation chromatography (GPC) tests were recorded on a Thermo ULTIMATE 3000 instrument. UV–vis spectra were measured on a Shimadzu UV3600 spectrophotometer. Photoluminescence spectra were measured on an Edinburgh photoluminescence FLS980 spectrometer. Transient absorption spectra were acquired on an Edinburgh LP980 laser flash photolysis spectrometer. ESR spectra were recorded on a Bruker EMXplus instrument. Morphologies of the nanoparticles were tested by FEI Tecnai G20 (TEM) operating at 200 kV. Dynamic light scattering (DLS) was measured on a Brookhaven Nanobrook Omni analyzer. The photoacoustic imaging (PAI) measurements were acquired on a Fujifilm VisualSonics Vevo LAZR 2100 multi‐modality PA imaging platform.

### Synthesis and Characterization

The synthetic procedure of DTPA‐S was presented in Scheme S1, Supporting Information. The synthesis of DAP was referred to as a reported procedure.^[^ ^41^, ^48^ ^]^

### Preparation of DTPA‐S

DAP (714.0 mg, 1.0 mmol) and *N,N*‐diphenyl‐4‐(4,4,5,5‐tetramethyl‐1,3,2‐dioxaborolan‐2‐yl)aniline (0.80 g, 2.2 mmol) were dissolved in 20 mL dimethylacetamide and placed in a 50 mL round‐bottled flask. The reaction flask was vacuumed and placed in an N_2_ atmosphere. Then, tetrakis(triphenylphosphine) palladium (0.10 g, 0.1 mmol) was added to the flask. After 10 min bubbling with N_2_, K_2_CO_3_ (0.40 g, 2.9 mmol) was dissolved in 2 mL deionized water and added into the reaction flask. The reactive solution was heated to 80 °C for 24 h. After the reaction completion, the resulting mixture was cooled and extracted twice with dichloromethane. The organic phase was collected, and the solvent was removed under vacuum. The residue obtained was purified by flash column chromatography (silica gel, *n*‐hexane: EtOAc = 40: 1) to give DTPA‐S as a purple‐black powder (yield: 0.70 g, 64.3%; purity: 97.88%). Anal. Calcd for C_66_H_66_N_4_S_4_: C, 75.97; H, 6.38; N, 5.37. Found: C, 75.89; H, 6.35; N, 5.42. ^1^H NMR (600 MHz, CDCl_3_) δ 8.11 (s, 2H), 7.57 (d, *J* = 8.6 Hz, 4H), 7.42 (d, *J* = 2.9 Hz, 2H), 7.30 (t, *J* = 7.9 Hz, 8H), 7.15 (d, *J* = 7.6 Hz, 8H), 7.11 – 7.04 (m, 8H), 3.59 – 3.54 (m, 4H), 1.88 – 1.79 (m, 2H), 1.56 – 1.50 (m, 4H), 1.48 – 1.44 (m, 4H), 1.43 – 1.41 (m, 4H), 1.40 – 1.34 (m, 4H), 0.99 (t, *J* = 7.4 Hz, 6H), 0.92 (t, *J* = 7.2 Hz, 6H).^13^C NMR (150 MHz, CDCl_3_) δ 161.78, 149.77, 148.55, 147.12, 139.67, 137.07, 129.46, 127.88, 126.94, 126.65, 125.00, 123.68, 123.47, 122.85, 107.95, 45.99, 39.23, 30.32, 28.52, 23.68, 23.12, 14.10, 10.62. MALDI‐TOF‐MS m/z calcd. for C_66_H_66_N_4_S_4_ [M+H]^+^ = 1043.4243, found = 1043.4247.

### Preparation of DTPA‐S‐1

DTPA‐S‐1 was synthesized by a similar procedure as DTPA‐S using DAP and 4‐(4,4,5,5‐Tetramethyl‐1,3,2‐dioxaborolan‐2‐yl)‐*N,N*‐bis(4‐methoxyphenyl)aniline as the starting materials to obtain 0.70 g black powder with the yield of 61.2%. Anal. Calcd for C_70_H_74_N_4_O_4_S_4_: C, 72.25; H, 6.41; N, 4.81. Found: C, 72.19; H, 6.37; N, 4.86. ^1^H NMR (600 MHz, CDCl_3_) δ 8.08 (s, 2H), 7.51 (d, *J* = 8.8 Hz, 4H), 7.37 (d, *J* = 4.1 Hz, 2H), 7.10 (d, *J* = 8.9 Hz, 8H), 6.91 (d, *J* = 8.8 Hz, 4H), 6.86 (d, *J* = 8.9 Hz, 8H), 3.81 (s, 12H), 3.59 – 3.50 (m, 4H), 1.85 – 1.82 (m, 2H), 1.54 – 1.51 (m, 4H), 1.49 – 1.46 (m, 4H), 1.45 – 1.39 (m, 4H), 1.37 – 1.34 (m, 4H), 0.99 (t, *J* = 7.4 Hz, 6H), 0.92 (t, *J* = 7.2 Hz, 6H).^13^C NMR (150 MHz, CDCl_3_) δ 163.47, 156.37, 149.33, 145.47, 137.34, 136.30, 134.02, 127.09, 126.89, 125.65, 124.77, 123.97, 119.71, 114.83, 55.53, 39.76, 36.37, 32.72, 29.04, 25.90, 23.08, 14.21, 11.09. MALDI‐TOF‐MS m/z calcd. for C_70_H_74_N_4_O_4_S_4_ [M+H]^+^ = 1163.4666, found = 1163.4659.

### Preparation of DTPA‐S‐3

DTPA‐S‐1 (0.50 g, 0.40 mmol) was dissolved in 20 mL of dichloromethane in a 50 mL three‐neck flask. A low‐temperature thermometer was inserted into the three‐neck flask, and the reaction flask was evacuated and placed in a nitrogen atmosphere. The reaction flask was then placed in an acetone bath and cooled to ‐78 °C with liquid nitrogen. After that, 2 mL of BBr_3_ was slowly added using a glass syringe, and the mixture was kept at ‐78 °C for 10 min before the acetone bath was removed and the reaction was allowed to proceed at room temperature for 2 h. The color of the reaction mixture changed from blue to black. After the reaction was completed, water and methanol were slowly added to the reaction flask to quench the reaction, then the solvent was evaporated under vacuum. The reaction mixture was extracted with ethyl acetate and water twice. The color of the organic phase changed from black to green. The organic phase was collected and dried over anhydrous sodium sulfate, and the solvent was then evaporated, affording DTPA‐S‐2, which was used without further purification. The residue (DTPA‐S‐2) was dissolved in 30 mL of acetone and placed in a 50 mL round‐bottom flask without further purification. 3‐Bromo‐1‐propyne (0.40 g, 3.0 mmol) and potassium carbonate (0.70 g, 5.0 mmol) were added to the reaction mixture and the reaction flask was evacuated and placed under a nitrogen atmosphere. Then, the mixed solution was heated to 80 °C and allowed to react continuously for 12 h. After the reaction was completed, the solvent was evaporated by rotary evaporation. The product was extracted twice with dichloromethane, and the organic phase was collected and dried with anhydrous sodium sulfate. The organic phase was concentrated and purified by column chromatography with a solvent system of petroleum ether and ethyl acetate in a volume ratio of 50:1 to give a dark green solid (0.30 g, yield: 59.1%). Anal. Calcd for C_78_H_74_N_4_O_4_S_4_: C, 74.37; H, 5.92; N, 4.45. Found: C, 74.32; H, 5.89; N, 4.49. ^1^H NMR (600 MHz, CDCl_3_) δ 8.06 (s, 2H), 7.52 (d, *J* = 7.7 Hz, 4H), 7.37 (s, 2H), 7.10 (d, *J* = 7.9 Hz, 8H), 6.94 (d, *J* = 7.8 Hz, 12H), 4.69 (s, 8H), 3.57 – 3.52 (m, 4H), 2.55 (s, 4H), 1.84 (s, 2H), 1.58 – 1.32 (m, 16H), 1.00 (t, *J* = 7.4 Hz, 6H), 0.92 (t, *J* = 7.2 Hz, 6H).^13^C NMR (150 MHz, CDCl_3_) δ 154.27, 151.51, 150.70, 149.03, 140.97, 137.46, 134.05, 131.63, 126.93, 126.85, 126.14, 124.11, 120.36, 115.94, 78.62, 75.64, 56.18, 39.78, 36.42, 32.73, 29.06, 25.91, 23.09, 14.23, 11.10. MALDI‐TOF‐MS m/z calcd. for C_78_H_74_N_4_O_4_S_4_ [M+H]^+^ = 1259.4666, found = 1259.4661.

### Preparation of NanoDTPA‐S/RGD

DTPA‐S‐3 (50.0 mg, 0.4 mmol), N_3_‐PEG_2000_‐RGD (0.20g, 0.2 mmol), copper(II) sulfate pentahydrate (50.0 mg, 0.2 mmol), and sodium ascorbate (79.0 mg 0.4 mmol) were dissolved in 5 mL DMSO under a nitrogen atmosphere. The reaction mixture was heated to 30 °C for 48 h. After the reaction was completed, the reaction mixture was transferred into a dialysis bag with a molecular weight cutoff of 3500 D against distilled water for 48 h to remove the unreacted chemicals. Then, the solution was lyophilized to give NanoDTPA‐S/RGD as a dark green solid (0.20 g).

### Preparation of NanoDTPA‐S

NanoDTPA‐S was prepared and dialyzed following a similar procedure as NanoDTPA‐S/RGD using mPEG_2000_‐N_3_ instead of N_3_‐PEG_2000_‐RGD as the starting material for the click reaction. NanoDTPA‐S was a dark green solid with the characteristic chemical shift of PEG at 3.63 ppm.

### High‐Performance Liquid Chromatography

HPLC chromatograms were acquired on a Waters e2695 system with a 2489 UV/Vis detector at a detection wavelength of 254 nm. Briefly, an ODS column (250 × 4.6 mm, 5 µm) was employed with a mobile phase of methanol/water for elution (40/60, v/v, 30min). DTPA‐S was dissolved in the mixed solution of methanol and water (40/60, v/v) at a concentration of 50 µm, and the injection volume was 20 µL.

### Gel Permeation Chromatography

GPC tests were recorded on Thermo ULTIMATE 3000 instruments with dual PL aquagel‐OH MIXED‐H columns (2 × 300 × 7.5 mm, 8 µm) and a UV detector at 254 nm. The GPC columns were eluted with water at 30 °C at the flow rate of 1mL min^−1^. NanoDTPA‐S/RGD were dissolved in PBS solution at the concentration of 50 µm, and the injection volume was 20 µL.

### Computational Details

DFT calculations were performed at the B3LYP/6‐31g (d, p) level with the SMD solvent model by using the Gaussian 16 package.^[^ ^54^, ^55^ ^]^ The reorganization energies of DTPA‐O and DTPA‐S between S_1_ and S_0_ states were calculated with a normal‐mode analysis approach using the DUSHIN program.^[^ ^56^ ^]^

### Photostability Experiment

DTPA‐S was dissolved in dichloromethane at a concentration of 10 µm. Absorption spectra of DTPA‐S were recorded every 5 min upon the irradiation of a 730 nm laser (0.3 W cm^−^^2^) for 30 min on a Shimadzu UV‐3600 spectrophotometer. The spectrum of ICG (5 µm) was measured as a reference under the same condition.

### Reactive Oxygen Species (ROS) Generation Capacity

The ROS generation ability of the compounds was evaluated under 730 nm laser irradiation. 1,3‐diphenyl‐isobenzofuran (DPBF) was used as a total ROS indicator, while 9,10‐anthracenediylbis(methylene)‐dimalonic acid (ABDA) and dihydroethidium (DHE) were used as the trapping agents of singlet oxygen (^1^O_2_) and O_2_˙ˉ, respectively. The time‐dependent absorption spectrum of the probes (DPBF, 20 µm: 250–550 nm, ABDA 20 µm: 300–500 nm) triggered by DTPA‐S (20 µm in dichloromethane) was recorded every 10 s with a 730 nm laser (0.3 W cm^−^^2^) irradiation. For the DHE probe, the fluorescence spectra (530‐800 nm with an excitation wavelength of 510 nm) of DHE (50 µm) in the presence of ctDNA (250 µg mL^−1^) and DTPA‐S encapsulated with pluronic F127 (20 µm) in water were recorded after every 10 s of 730 nm laser irradiation (0.3 W cm^−2^). For the ROS quantum yield measurement, ICG was used as a reference (*Φ*_Δ_ = 0.14).^[^ ^57^ ^]^

### Electron Spin Resonance Measurements

The ESR spectra were recorded on a Bruker EMXplus instrument spectrometer at 3.17 mW microwave power, 100 G SweepWidth, and 1G field modulation. 2,2,6,6‐Tetramethyl‐4‐piperidone (TEMP), and 5,5‐dimethyl‐ 1‐pyrroline N‐oxide (DMPO) were used as the spin‐trapping agents. Mixed solutions of NanoDTPA‐S/RGD (50 µm) and TEMP (20 mm, water) or DMPO (20 mm, methanol) were under 880 nm laser irradiation (0.3 W cm^−2^), and the electron spin resonance spectra were recorded.

### Transient Absorption Spectroscopy

The transient absorption spectra were measured on an LP980 laser flash photolysis spectrometer (Edinburgh Instruments, UK). The signal was digitized with a Tektronix TDS 3012B oscilloscope. The samples were dissolved in deaerated dichloromethane at a concentration of 50 µm and excited with a nanosecond pulsed laser (Surelite I‐10, USA). The excitation wavelength was 690 nm with a laser energy of 45 mJ per pulse.

### Photothermal Conversion Performance

The photothermal performance of NanoDTPA‐S/RGD was assessed by exposing various concentrations of 0.5 mL NanoDTPA‐S/RGD aqueous solution (0.0, 0.2, 0.4, 0.8, and 1.2 mg mL^−1^) to a laser at 880 nm (0.3 W cm^−^^2^). The temperature change of the solutions during laser irradiation was recorded using a thermal imaging instrument. Each sample was tested for a duration of 10 min. The photostability of NanoDTPA‐S/RGD was evaluated by monitoring the real‐time temperature of 0.5 mL aqueous solution (1.2 mg mL^−1^) during 880 nm laser irradiation (0.3 W cm^−^^2^) over 5 cycles of heating/cooling processes. ICG aqueous solution at a concentration of 10 µm was tested using a similar procedure as a control.

### Cell Lines and Cell Culture

Human non‐small cell lung cancer cells A549 were provided by KeyGEN Biotec Co., LTD (Nanjing, China, cat. No. KG007). The cells were grown monolayer using high glucose Dulbecco's modified Eagle medium (DMEM, KeyGEN Biotec, cat. No. KGM12800N‐500) with 10% fetal bovine serum (FBS, KeyGEN Biotec, cat. No. KGY009), streptomycin (100 µg mL^−1^) and penicillin (100 µg mL^−1^) solutions. The cells were cultured in a humidified atmosphere containing 5% CO_2_ at 37 °C at a density of 1 × 10^4^ viable cells cm^−2^, approximately. Similar procedures were carried out inside a hypoxic chamber (2% O_2_, 37.0 °C) for hypoxic experiment.

### In Vitro Photocytotoxicity Evaluation (MTT Assays)

For normoxic conditions, A549 cells in the logarithmic growth phase were placed into a 96‐well plate at a density of 2×10^5^ cells/well and then incubated in a cell culture incubator (5% CO_2_, 95% air, 37.0 °C) for 12 h. Various concentrations of NanoDTPA‐S/RGD (100, 20, 4, 0.8, 0.16, and 0.032 µm) were added to the plate and incubated for another 24 h. Subsequently, the cells were exposed to an 880 nm laser (0.3 W cm^−^^2^) for 10 min and then continued to incubate in the dark for another 24 h for the laser irradiation group. For the dark group, the cells were kept in the dark for 48 h after NanoDTPA‐S/RGD was added. After incubation, the cells were stained with 10 µL of MTT (0.5 mg mL^−1^) for 4–5 h. Finally, all the culture media were removed from the wells and 200 µL of DMSO were added. The O.D. values were measured using a 490/570 nm enzyme‐labeling instrument. The half‐inhibitory concentration (IC_50_) values of the samples were determined from the resulting dose‐dependence curves by SPSS software. The experiment was carried out parallel at least three times. For the hypoxic experiment, similar procedures were operated inside a hypoxic chamber (2% O_2_, 37.0 °C).

### Live/Dead Cell Co‐Staining Assay

A549 cells were treated with 40 µm of NanoDTPA‐S/RGD. After 24 h incubation under hypoxia or normoxia conditions, the cells were irradiated with 880 nm (0.3 W cm^−2^) laser irradiation for 10 min. The cells were further incubated for another 24 h in the dark, and then stained with calcein AM (5 mm) and propidiumiodide (5 mm) for 30 min, and washed with PBS (10 mm, pH 7.4). Finally, the images of the stained cells were captured using a confocal microscope.

### Laboratory Animals

The in vivo experimental protocols were in accordance with the regulations of the Animal Care & Welfare Committee of Southeast University (Nanjing, China, permit no. SYXK2021‐0022) and KeyGEN BioTECH Co. Ltd. (Nanjing, China, permit no. SYXK‐20170040). BALB/c nude mice (female, 5–6 weeks, 18–20 g) were used as animal models in this research. The animals were hosted in an equipped animal facility with a temperature of 20–26 °C.

### In Vivo Experiment

For the construction of tumor‐bearing model mice, 0.1 mL of PBS with 2 × 10^7^ A549 cells were injected into the armpit of each BALB/c nude mice (female, 5–6 weeks, 18–20 g). The mice were randomized into 4 groups with 4 mice in each group when the tumors grew to a volume of 100 mm^3^ approximately. The animals were subjected to intravenous injection through the tail vein with NanoDTPA‐S/RGD (2.0 mg kg^−1^) on days 1, 7, and 14. Then, the tumor sites were irradiated with 880 nm (0.3 W cm^−2^, 10 min) laser at 4 h post injection. The tumor volumes and body weights of the mice were measured every 3 days. Tumor volumes were obtained by measuring the perpendicular diameter of the tumor in length and width and calculated according to the formula: Tumor volume (mm^3^) = 1/2 × length × width^2^. Afterward, the mice were sacrificed and the tumor tissues and major organs of the mice were harvested for histological examinations.

### H&E Staining

On the 21^st^ day, the mice were sacrificed, and the tumor tissue and major organs (heart, liver, spleen, lungs, kidneys) were harvested. The tissue samples were dried and embedded in paraffin and dewaxed in xylene, rehydrated by gradient ethanol, washed with distilled water, and then stained with hematoxylin and eosin (H&E). After staining, the sections were dehydrated using an increasing concentration of ethanol and xylene. The tissue morphology was observed with a light microscope.

### PA Imaging

The in vitro PA signals of NanoDTPA‐S and NanoDTPA‐S/RGD at various concentrations (0, 1, 2, 4 mg mL^−1^) in 0.26 × 0.61 mm polyethylene capillaries were collected with the excitation wavelength of 880 nm. To investigate in vivo PA imaging, A549 tumor (appr. 200 mm^3^) bearing BALB/c nude female mice were anesthetized by isoflurane gas before PA imaging. The in vivo PA images and signal intensities at tumor sites were recorded from 0 to 24 h of post‐injection through the tail veil with NanoDTPA‐S and NanoDTPA‐S/RGD (2 mg kg^−1^) with an excitation wavelength of 880 nm.

### Statistical Analysis

All data were presented as mean ± s.d. unless otherwise specified. A two‐sided student's test was used to evaluate the statistical significance. *p*‐Values < 0.05 represented statistical significance, *p*‐Values: ***p < 0.001 (unpaired, two‐sided t‐tests).

## Conflict of Interest

The authors declare that they have no conflict of interest.

## Supporting information



Supporting Information

## Data Availability

The data that support the findings of this study are available in the supplementary material of this article.
