# Supplementary material for: Molecular Engineering of a Tumor‐Targeting Thione‐Derived Diketopyrrolopyrrole Photosensitizer to Attain NIR Excitation Over 850 nm for Efficient Dual Phototherapy
Source: Adv Sci (Weinh). 2024 Oct 16;11(45):2407727. doi: 10.1002/advs.202407727 (PMC11615798; doi:10.1002/advs.202407727)
Supplement: Supplementary file 1 — Supporting Information [file ADVS-11-2407727-s001.pdf]

ADVANCED SCIENCE Open Access

Supporting Information

for *Adv. Sci.*, DOI 10.1002/advs.202407727

Molecular Engineering of a Tumor-Targeting Thione-Derived Diketopyrrolopyrrole Photosensitizer to Attain NIR Excitation Over 850 nm for Efficient Dual Phototherapy

Gang Xu, Yunxia Song, Haifeng Jin, Pengmin Shi, Yubo Jiao, Fangzhou Cao, Jie Pang, Yanyan Sun, Lei Fang, Xing-Hua Xia* and Jian Zhao**

Supporting Information

Molecular Engineering of a Tumor-Targeting Thione-Derived Diketopyrrolopyrrole Photosensitizer to Attain NIR Excitation Over 850 nm for Efficient Dual Phototherapy

Gang Xu,[a] Yunxia Song,[a] Haifeng Jin,[a] Pengmin Shi,[a] Yubo Jiao,[a] Fangzhou Cao,[a] Jie Pang,[b] Yanyan Sun,[c] Lei Fang,*[a] Xing-Hua Xia,*[b] Jian Zhao*[a]

[a] G. Xu, Y. Song, H. Jin, P. Shi, Y. Jiao, F. Cao, L. Fang, J. Zhao
Jiangsu Province Hi-Tech Key Laboratory for Biomedical Research, School of Chemistry and Chemical Engineering, Southeast University, Nanjing 211189, China
E-mail: lei.fang@seu.edu.cn, zhaojianzhaokuan@163.com

[b] J. Pang, X.-H. Xia,
State Key Lab of Analytical Chemistry for Life Science, School of Chemistry and Chemical Engineering, Nanjing University, Nanjing 210023, China
E-mail: xhxia@nju.edu.cn

[c] Y. Sun
School of Chemistry and Life Sciences, Suzhou University of Science and Technology, Suzhou 215009, China

Table of contents

Supplementary Schemes and Figures

Scheme S1. Synthetic route of DTPA-S.
Scheme S2. Synthetic route of NanoDTPA-S.

Figure S1. ^{1}H NMR spectrum of DTPA-S.
Figure S2. ^{13}C NMR spectrum of DTPA-S.
Figure S3. MALDI-TOF-MS diagram of DTPA-S.
Figure S4. HPLC chromatogram of DTPA-S.
Figure S5. The emission decay curve of DTPA-O in dichloromethane.
Figure S6. Time-dependent absorption spectra of (A) ICG in water (5 μM) and (B) DTPA-S in dichloromethane (10 μM) upon 730 nm (0.3 W/cm^2) laser irradiation. (C) Changes of the absorption intensity of ICG and DTPA-S at 779 and 712 nm, respectively.
Figure S7. Photocatalytic degradation of ABDA triggered by DTPA-S in dichloromethane under 730 nm laser irradiation (0.3 W cm^{-2}).
Figure S8. (A) Absorption spectra of DPBF in the presence of DTPA-O (40 μM) under 635 nm (0.3 W/cm^2) laser irradiation in dichloromethane. (B) Photocatalytic degradation of ABDA triggered by DTPA-O (40 μM) in dichloromethane under 635 nm laser irradiation (0.3 W cm^{-2}). (C) Time-dependent fluorescence spectra of DHE (50 μM) in the presence of ctDNA (250 μg mL^{-1}) and DTPA-O (40 μM) encapsulated with pluronic F127 in water under 635 nm laser irradiation (0.3 W/cm^2).
Figure S9. ^{1}H NMR spectrum of DTPA-S-1.
Figure S10. ^{13}C NMR spectrum of DTPA-S-1.
Figure S11. MALDI-TOF-MS diagram of DTPA-S-1.
Figure S12. ^{1}H NMR spectrum of DTPA-S-3.
Figure S13. ^{13}C NMR spectrum of DTPA-S-3.
Figure S14. MALDI-TOF-MS diagram of DTPA-S-3.
Figure S15. MALDI-TOF-MS diagram of P(DTPA-S/RGD).
Figure S16. ^{1}H NMR spectrum of P(DTPA-S/RGD).
Figure S17. GPC chromatogram of NanoDTPA-S/RGD.
Figure S18. UV-vis absorption spectra of NanoDTPA-S/RGD in PBS (A) pH=5.4, (B) pH=7.4, (C) pH=9.0, (D) FBS, and (E) DMEM at various time points.
Figure S19. The absorption spectra of DPBF in the presence of NanoDTPA-S/RGD under 880 nm (0.3 W/cm^2) laser irradiation in water.
Figure S20. Photocatalytic degradation of ABDA triggered by NanoDTPA-S/RGD under 880 nm laser irradiation (0.3 W cm^{-2}).
Figure S21. ESR signals of TEMPO and DMPO-OOH adducts for (A) $^1O_2$ and (B) $O_2^{\bullet-}$ characterization upon NIR (880 nm, 0.3 W/cm^2) irradiation of the mixture of NanoDTPA-S/RGD (50 μM) and TEMP (20 mM, water) or DMPO (20 mM, methanol) at 0 and 2 min, respectively.
Figure S22. Temperature elevation of NanoDTPA-S/RGD and ICG during five cycles of heating-cooling processes.
Figure S23. Relative cell viability of A549 cells treated with NanoDTPA-S/RGD at various concentrations under (A) normoxia and (B) hypoxia with Vc under laser irradiation.

Figure S24. Flow cytometry analysis of ROS levels in A549 cells after (A) normoxic, and (B) hypoxic treatment conditions.

Figure S25. (A) TEM image and (B) DLS profile of NanoDTPA-S.

Figure S26 *In vitro* PA signals of the NanoDTPA-S/RGD and NanoDTPA-S under 880 nm illumination.

Figure S27. *In vivo* PA images of the NanoDTPA-S/RGD-treated A549 tumor-bearing mice recorded under 880 nm illumination.

Figure S28. Tumor images from different groups of mice after 21 days' treatment.

Figure S29. Photos of sacrificed mice from different groups after 21 days' treatment.

Figure S30. Body weight of different groups of mice.

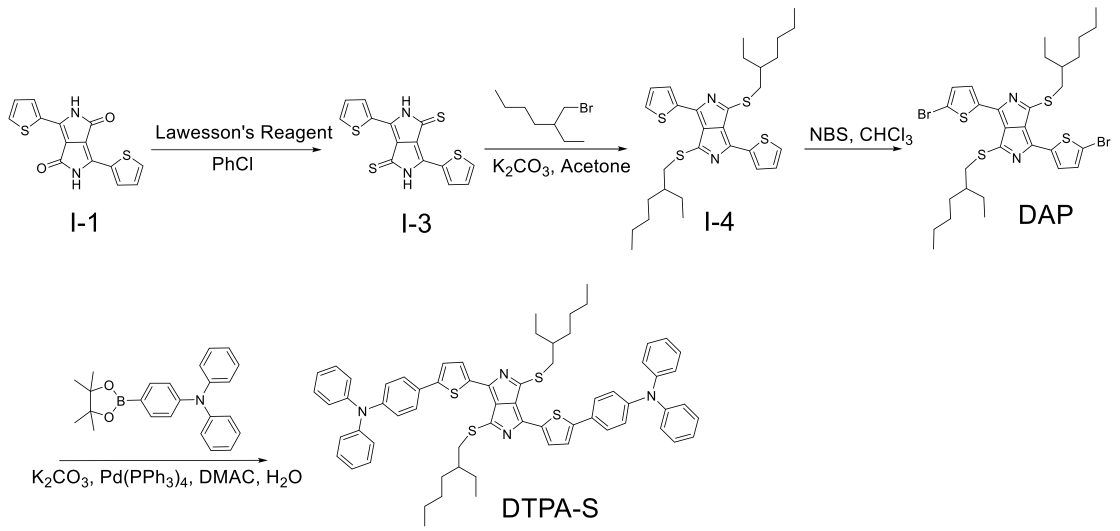

Scheme S1. Synthetic route of DTPA-S.

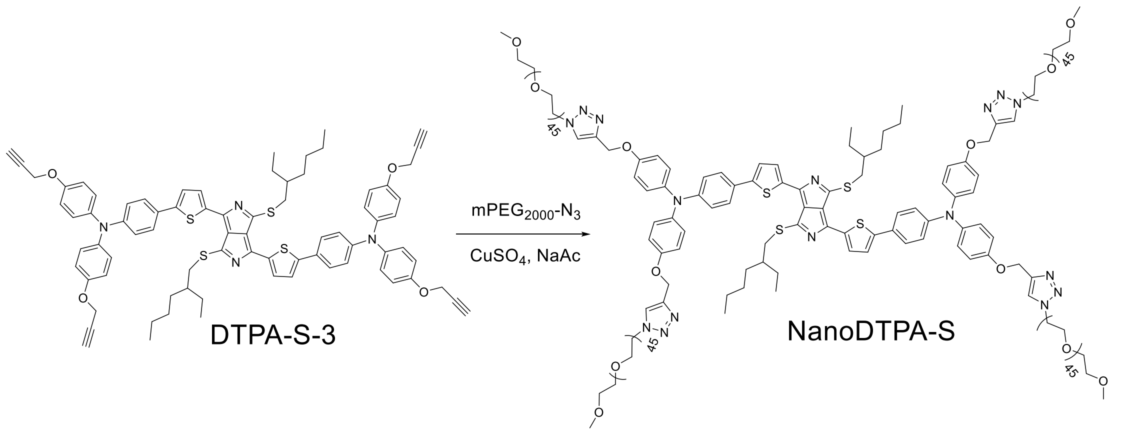

Scheme S2. Synthetic route of NanoDTPA-S.

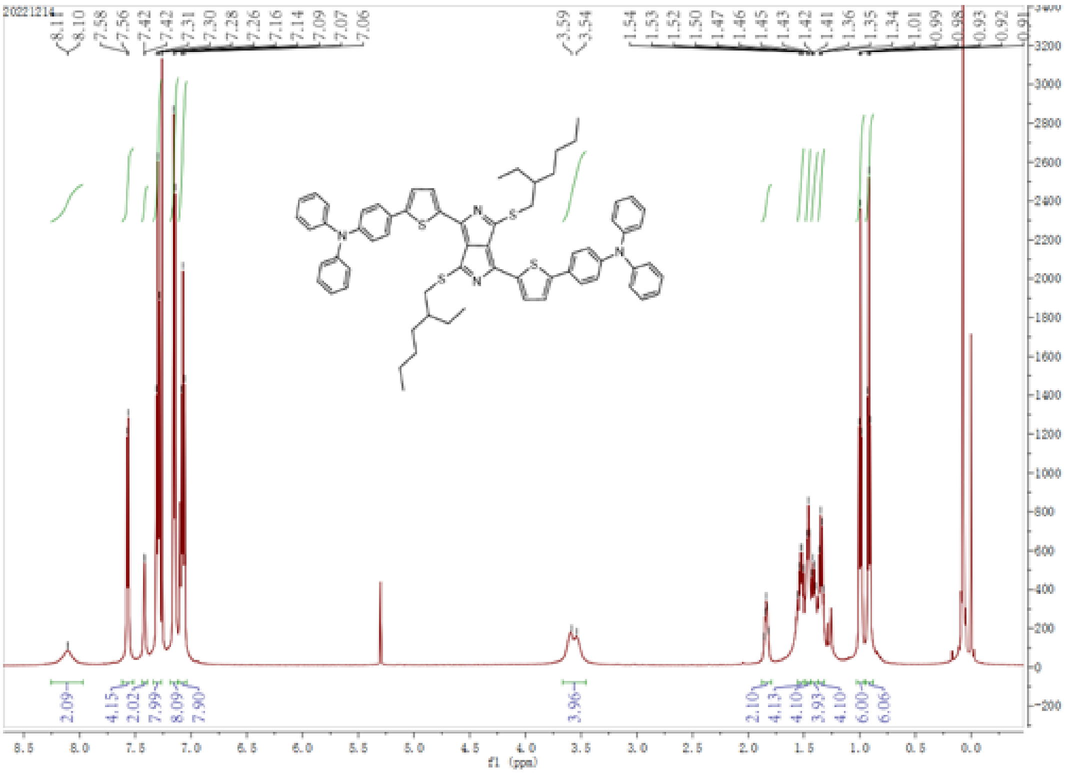

Figure S1. ^{1}H NMR spectrum of DTPA-S.

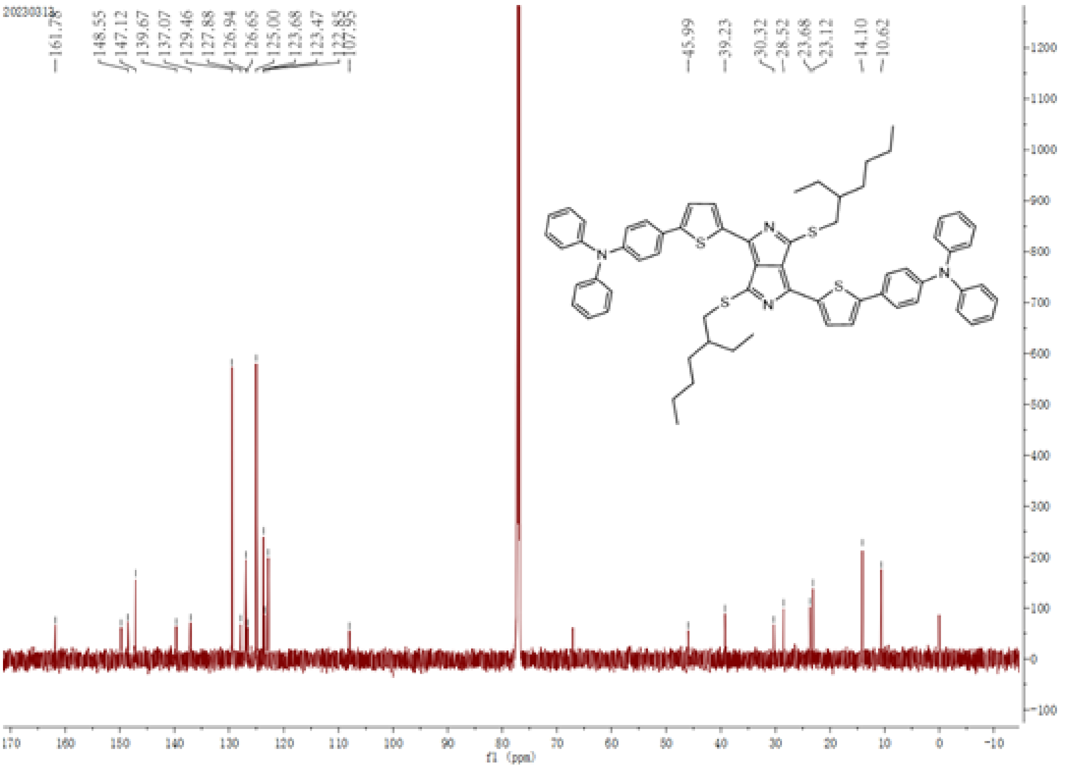

Figure S2. ^{13}C NMR spectrum of DTPA-S.

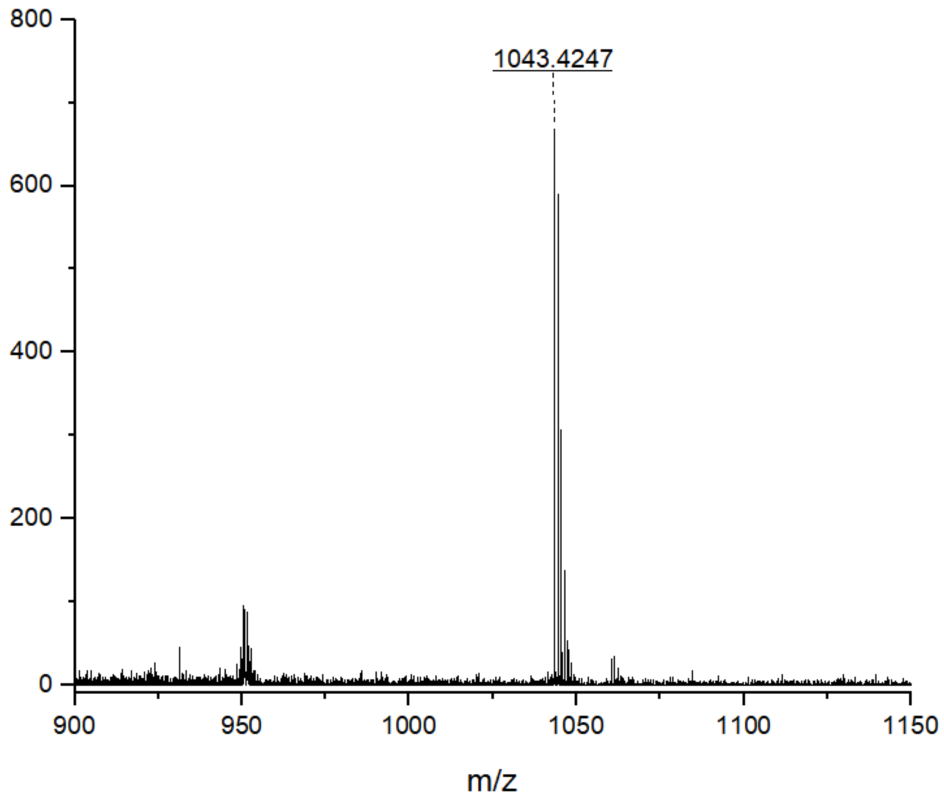

Figure S3. MALDI-TOF-MS diagram of DTPA-S.

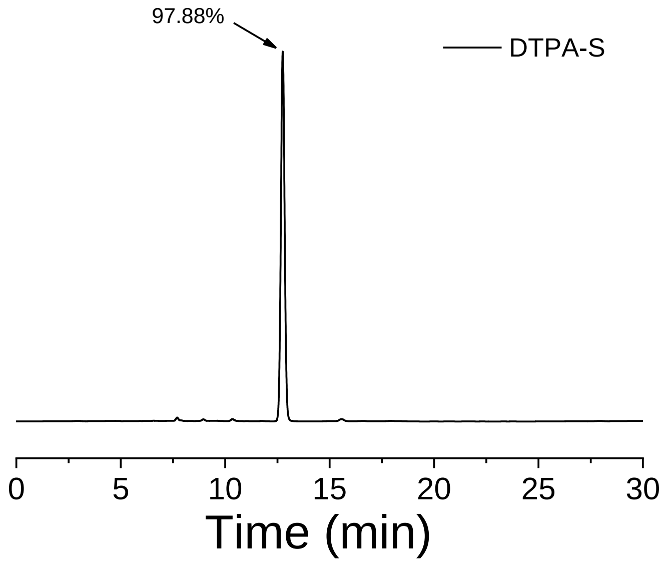

Figure S4. HPLC chromatogram of DTPA-S.

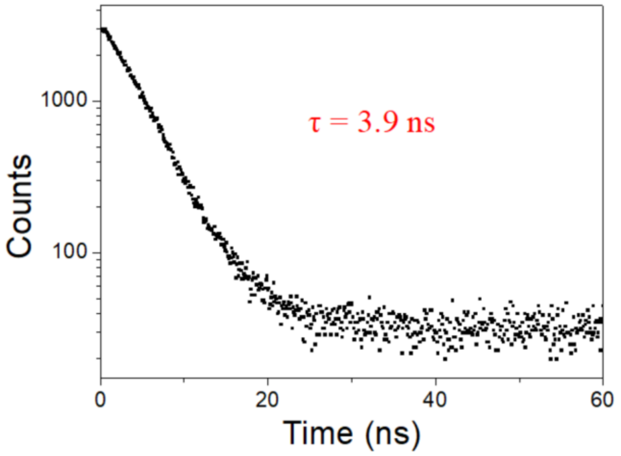

Figure S5. Emission decay curve of DTPA-O in dichloromethane.

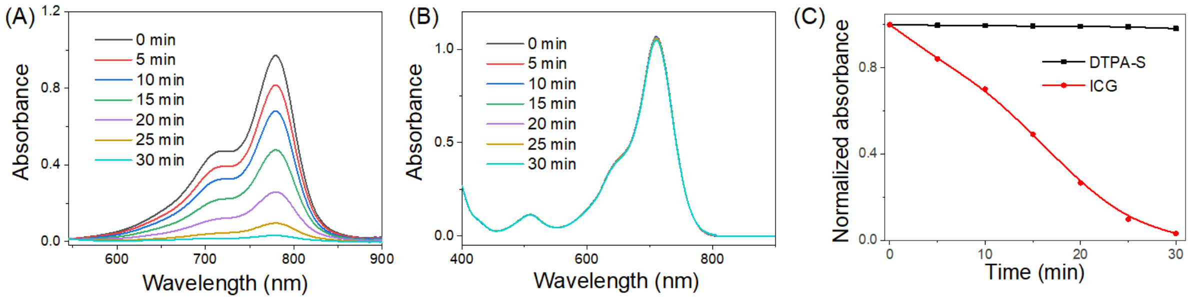

Figure S6. Time-dependent absorption spectra of (A) ICG in water (5 μM) and (B) DTPA-S in dichloromethane (10 μM) upon 730 nm (0.3 W/cm^2) laser irradiation. (C) Changes of the absorption intensity of ICG and DTPA-S at 779 and 712 nm, respectively.

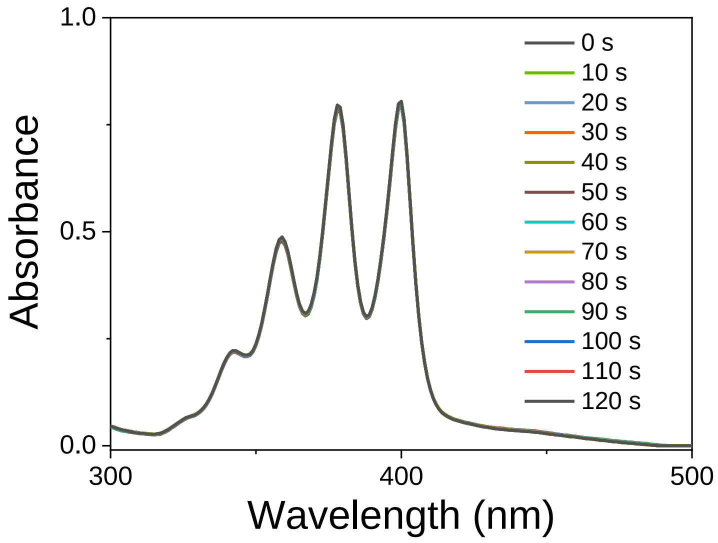

Figure S7. Photocatalytic degradation of ABDA triggered by DTPA-S in dichloromethane under 730 nm laser irradiation (0.3 W cm^{-2}).

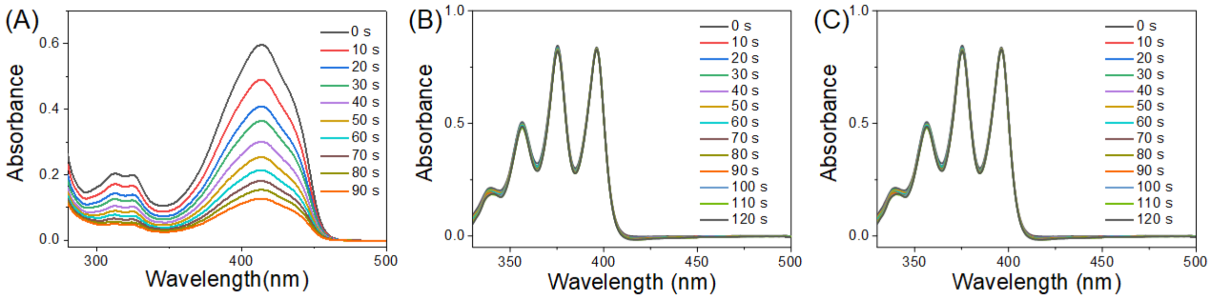

Figure S8. (A) Absorption spectra of DPBF in the presence of DTPA-O (40 µM) under 635 nm (0.3 W/cm^2) laser irradiation in dichloromethane. (B) Photocatalytic degradation of ABDA triggered by DTPA-O (40 µM) in dichloromethane under 635 nm laser irradiation (0.3 W cm^{-2}). (C) Time-dependent fluorescence spectra of DHE (50 µM) in the presence of ctDNA (250 µg mL^{-1}) and DTPA-O (40 µM) encapsulated with pluronic F127 in water under 635 nm laser irradiation (0.3 W/cm^2).

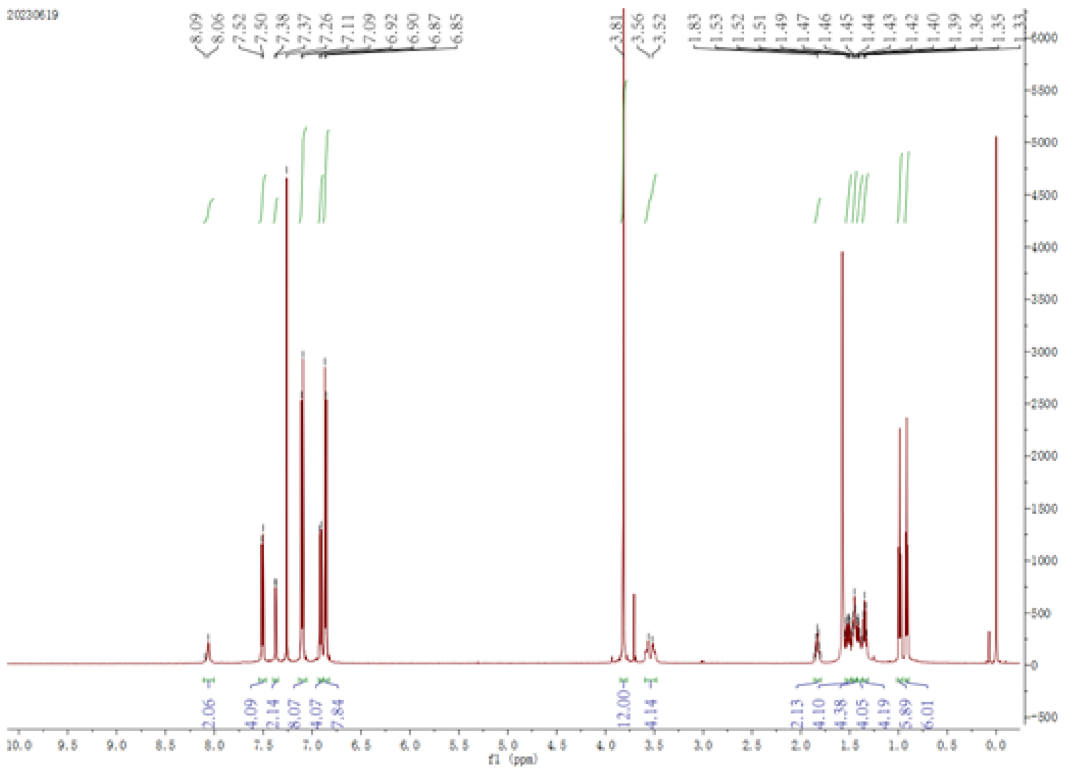

Figure S9. ^{1}H NMR spectrum of DTPA-S-1.

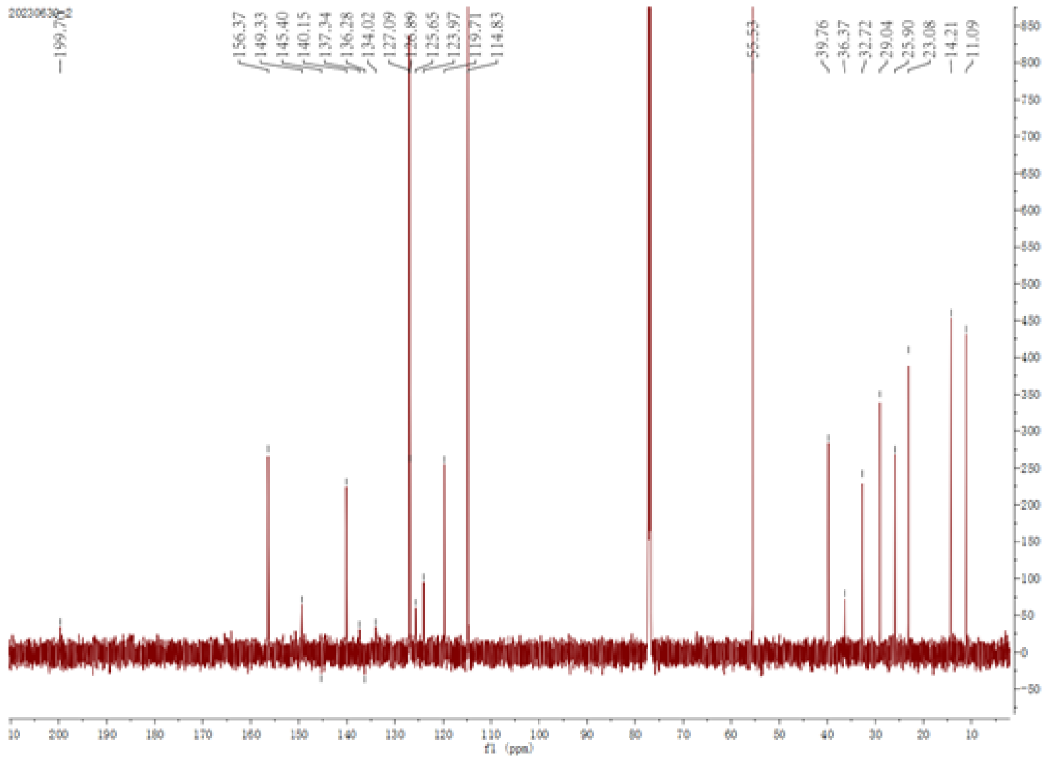

Figure S10. ^{13}C NMR spectrum of DTPA-S-1.

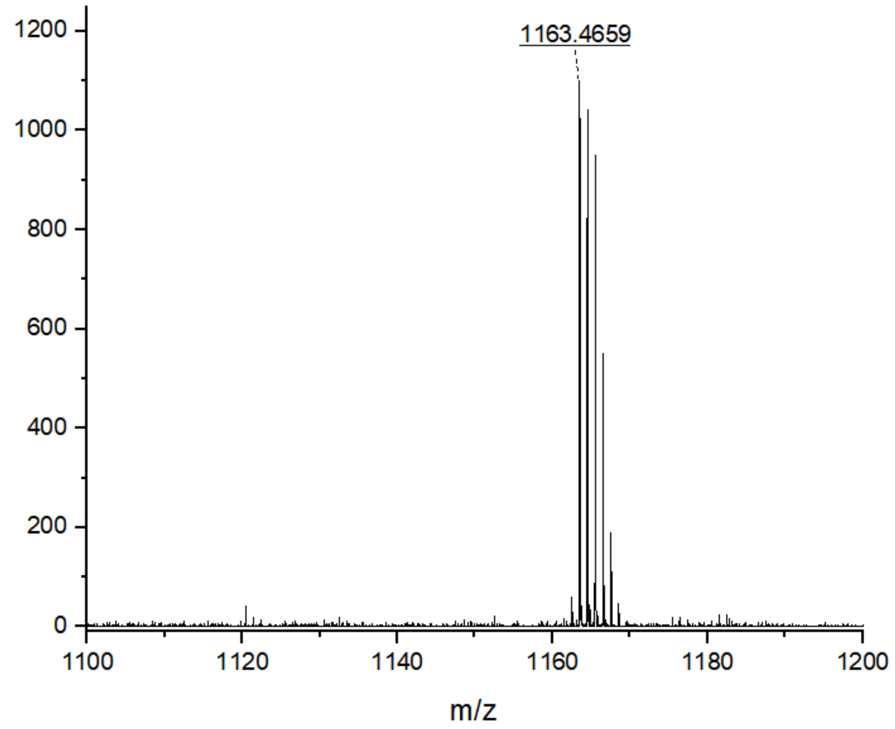

Figure S11. MALDI-TOF-MS diagram of DTPA-S-1.

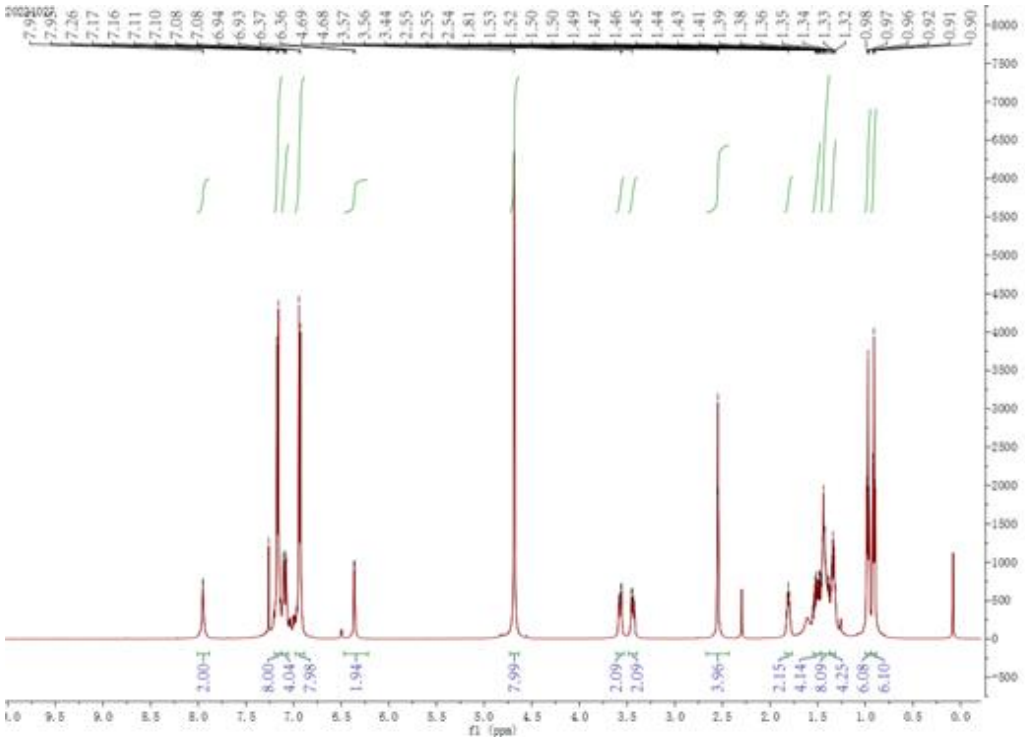

Figure S12. ^{1}H NMR spectrum of DTPA-S-3.

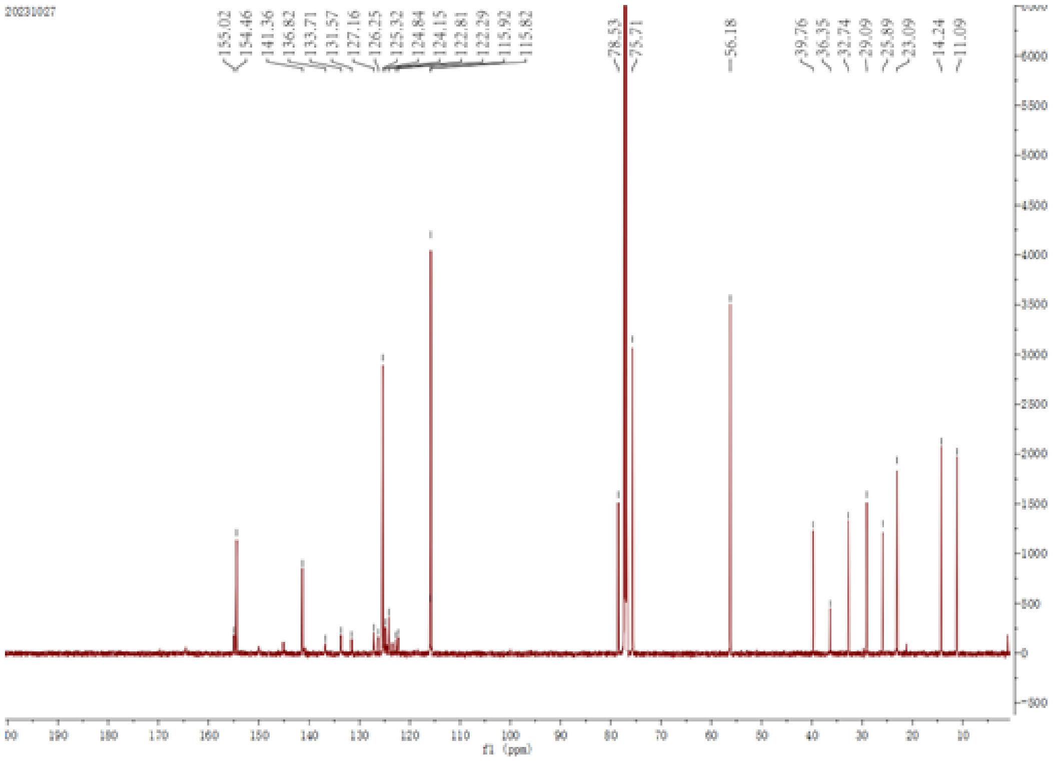

Figure S13. ^{13}C NMR spectrum of DTPA-S-3.

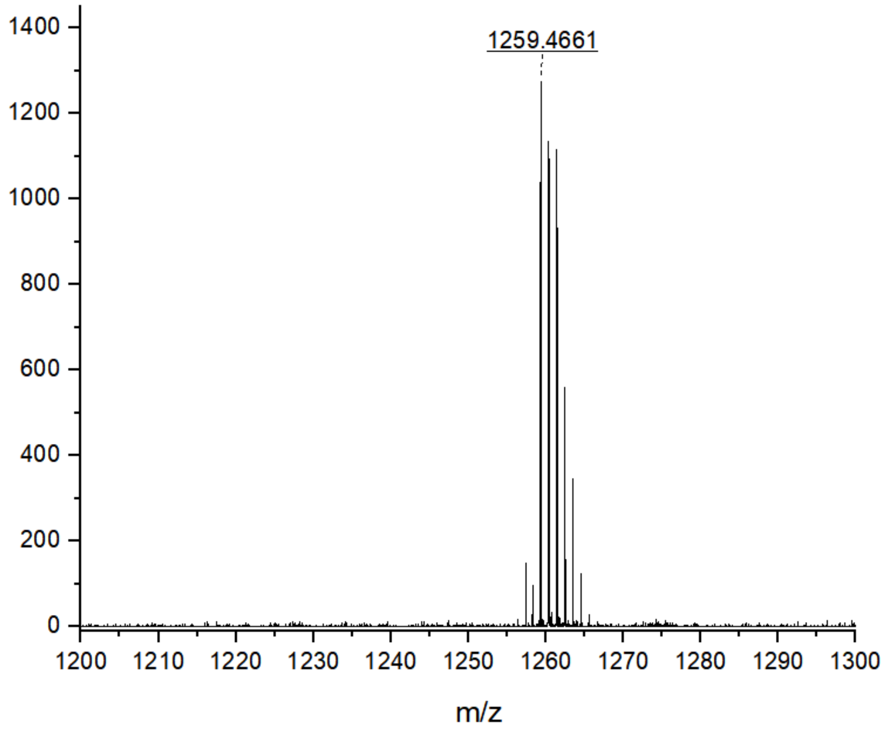

Figure S14. MALDI-TOF-MS diagram of DTPA-S-3.

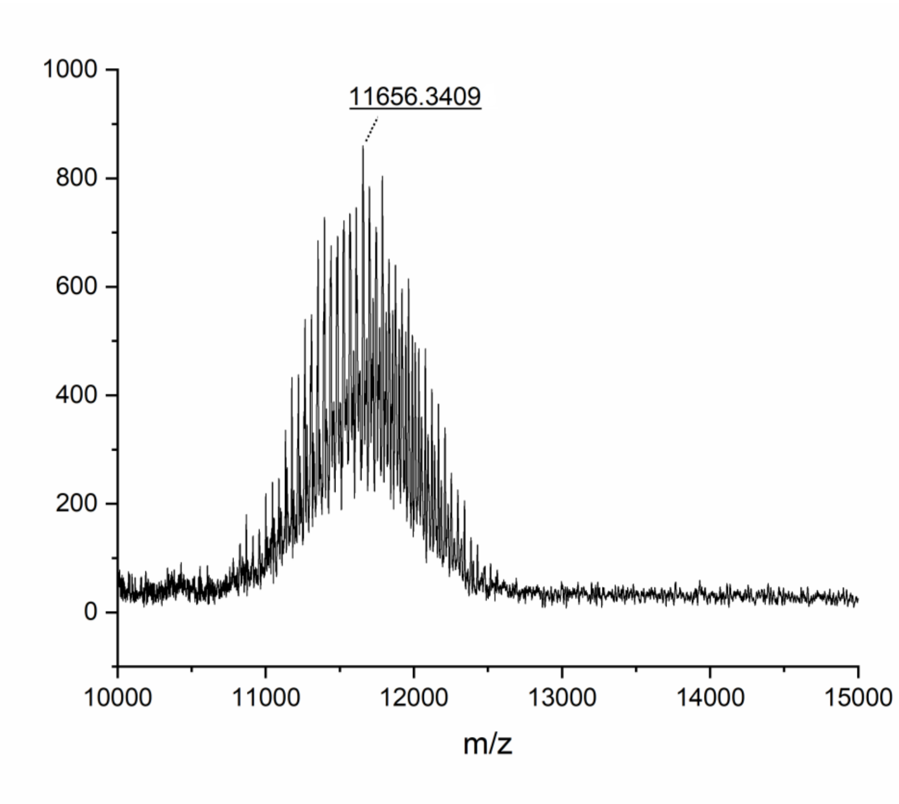

Figure S15. MALDI-TOF-MS diagram of P(DTPA-S/RGD).

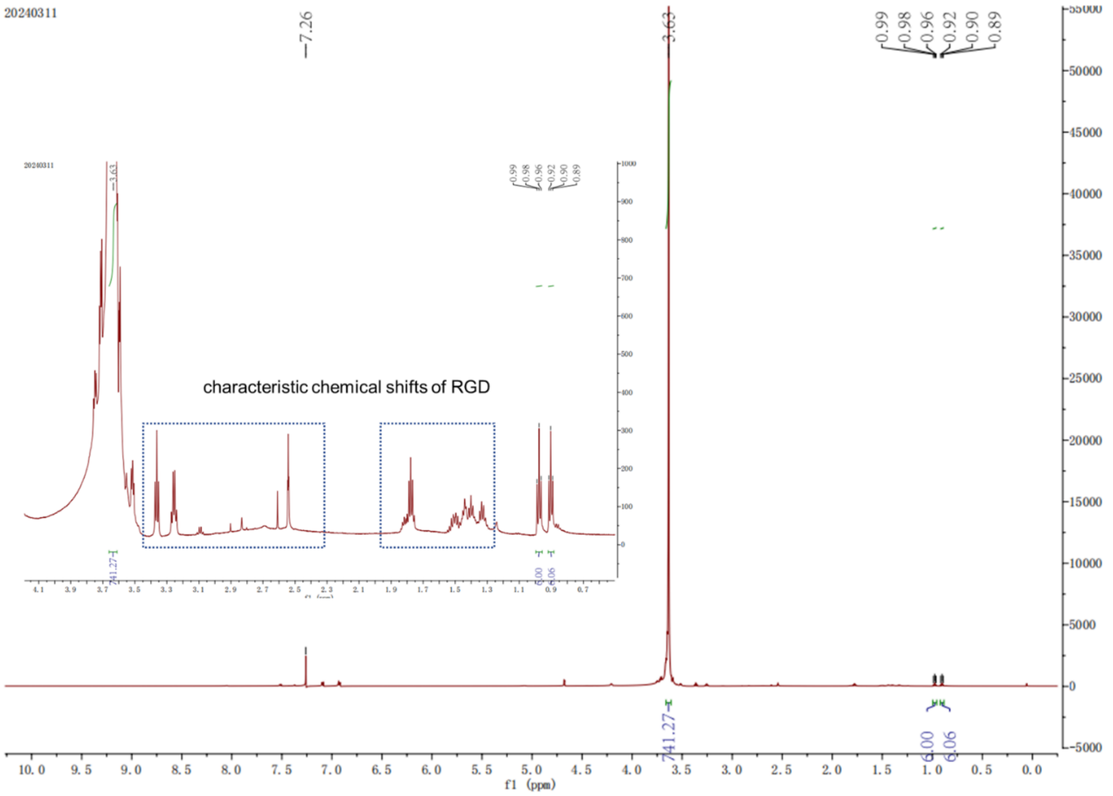

Figure S16. ^{1}H NMR spectrum of P(DTPA-S/RGD).

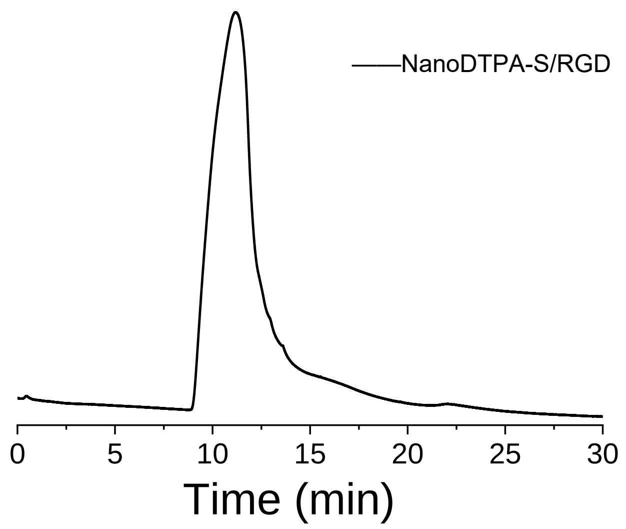

Figure S17. GPC chromatogram of NanoDTPA-S/RGD.

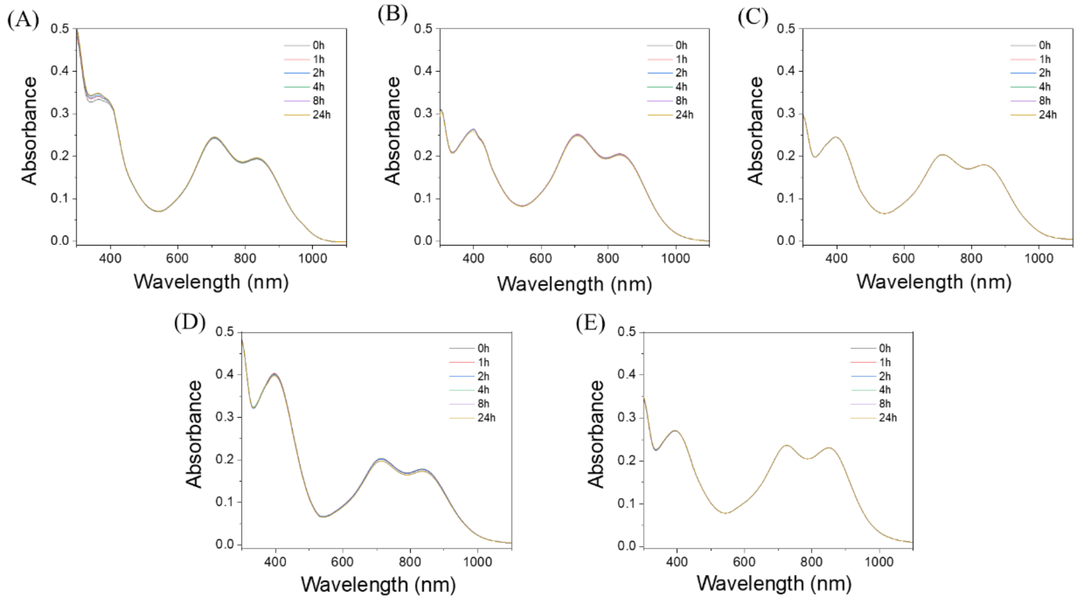

Figure S18. UV-vis absorption spectra of NanoDTPA-S/RGD in PBS (A) pH=5.4, (B) pH=7.4, (C) pH=9.0, (D) FBS, and (E) DMEM at various time points.

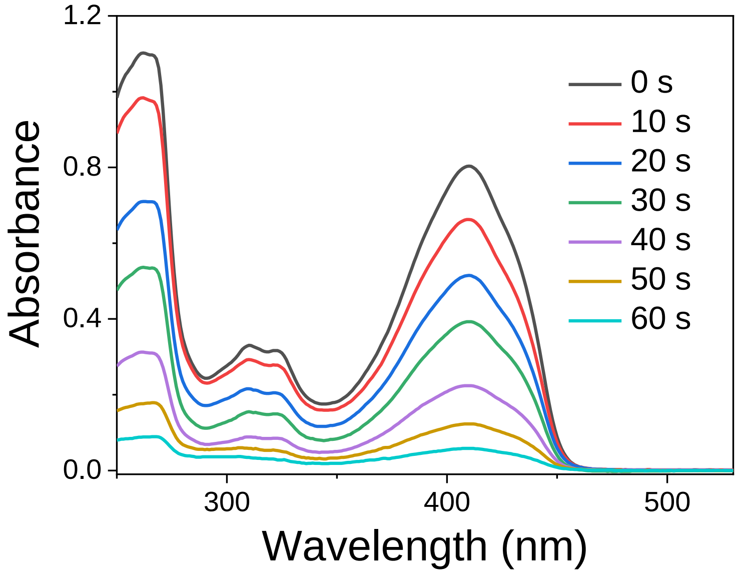

Figure S19. Absorption spectra of DPBF in the presence of NanoDTPA-S/RGD under 880 nm (0.3 W/cm^2) laser irradiation in water.

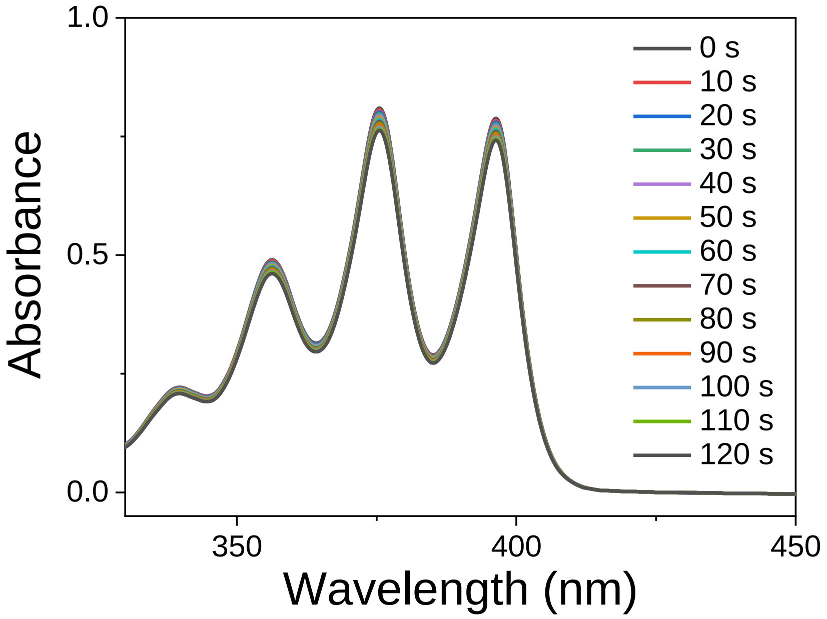

Figure S20. Photocatalytic degradation of ABDA triggered by NanoDTPA-S/RGD under 880 nm laser irradiation (0.3 W cm^{-2}).

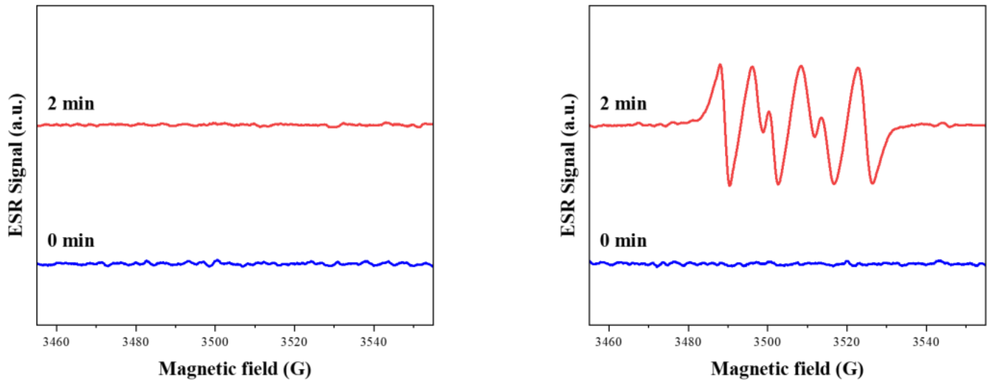

Figure S21. ESR signals of TEMPO and DMPO-OOH adducts for (A) $^1O_2$ and (B) $O_2^{\bullet-}$ characterization upon NIR (880 nm, 0.3 W/cm^2) irradiation of the mixture of NanoDTPA-S/RGD (50 μM) and TEMP (20 mM, water) or DMPO (20 mM, methanol) at 0 and 2 min, respectively.

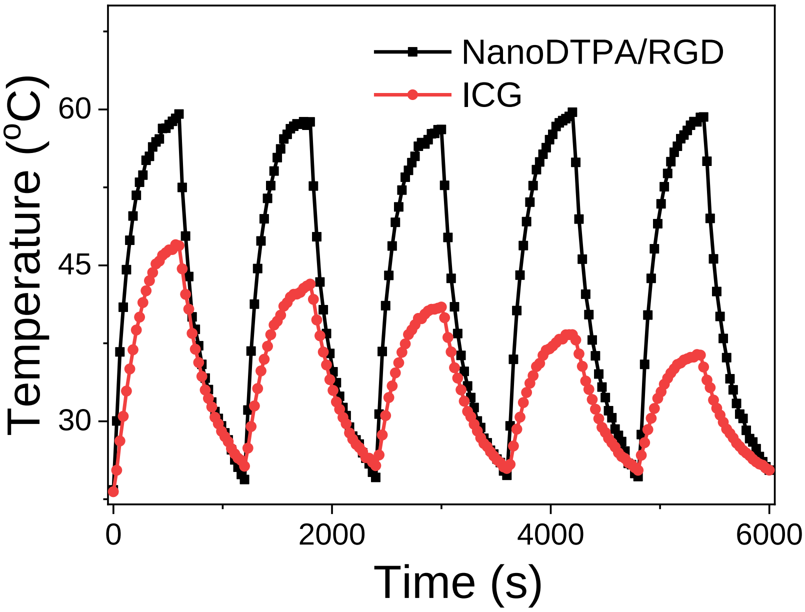

Figure S22. Temperature elevation of NanoDTPA-S/RGD and ICG during five cycles of heating-cooling processes.

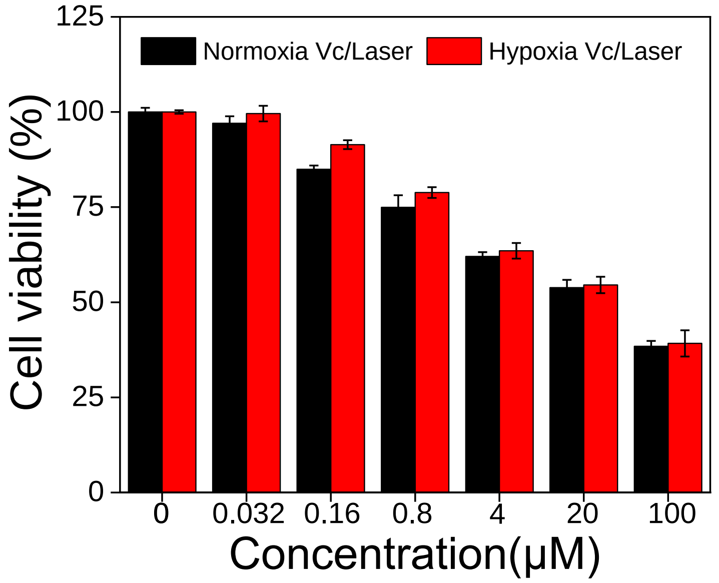

Figure S23. Relative cell viability of A549 cells treated with NanoDTPA-S/RGD at various concentrations under (A) normoxia and (B) hypoxia with Vc under laser irradiation.

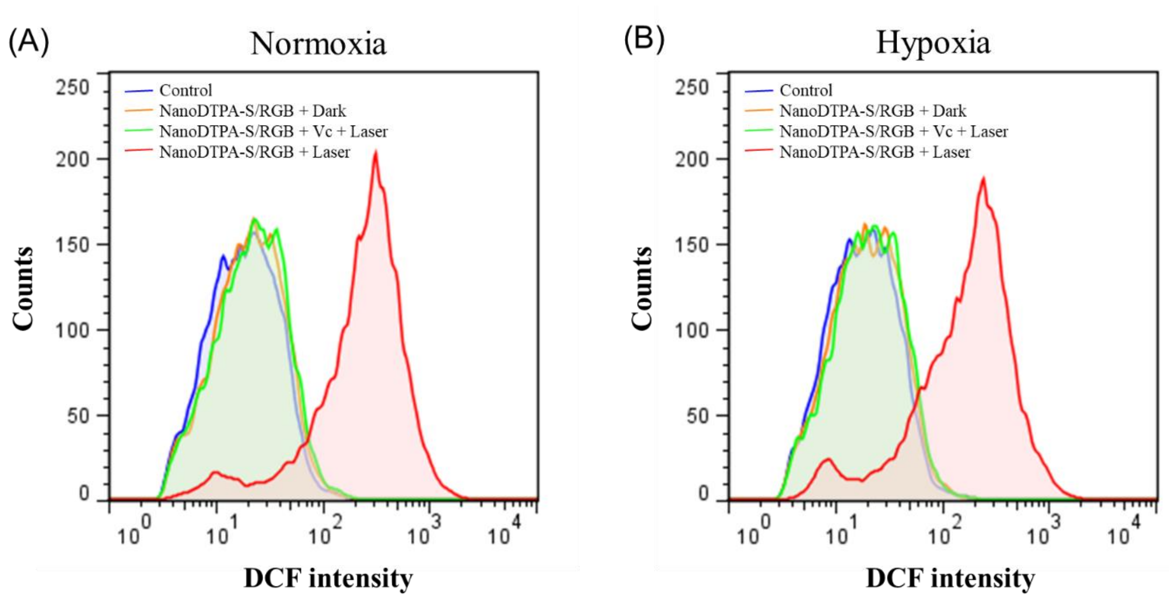

Figure S24. Flow cytometry analysis of ROS levels in A549 cells after (A) normoxic, and (B) hypoxic treatment conditions.

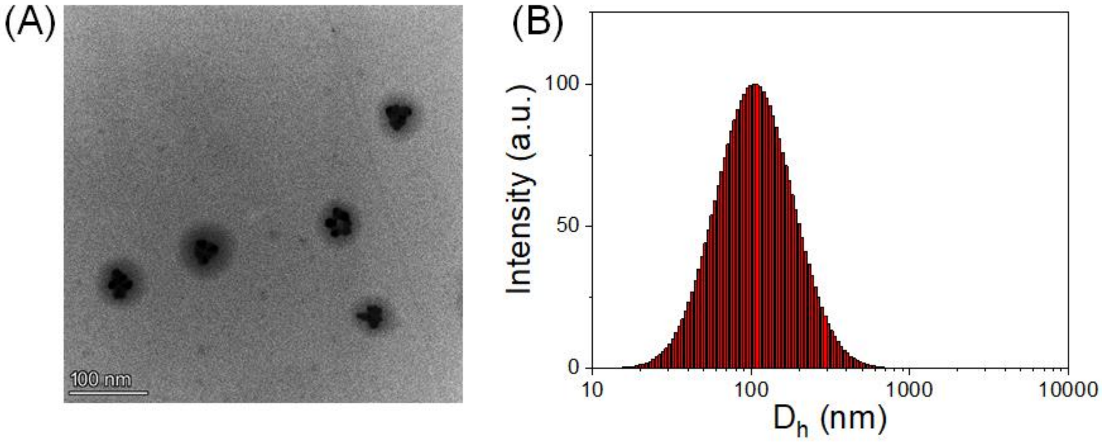

Figure S25. (A) TEM image and (B) DLS profile of NanoDTPA-S.

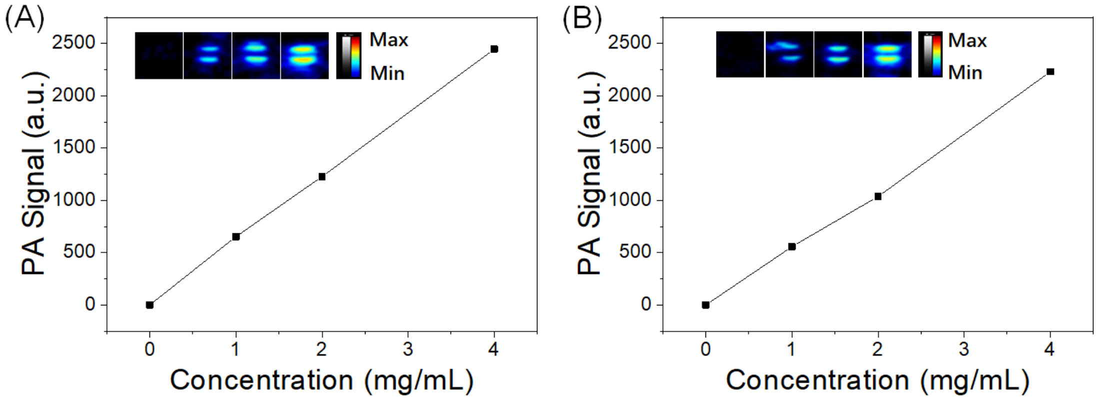

Figure S26. *In vitro* PA signals of (A) NanoDTPA-S/RGD and (B) NanoDTPA-S at different concentrations under 880 nm illumination.

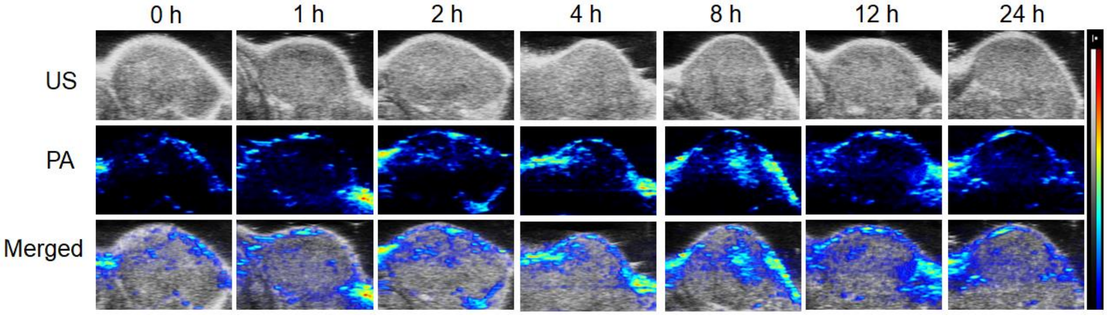

Figure S27. *In vivo* PA images of the NanoDTPA-S treated A549 tumor-bearing mice recorded under 880 nm illumination.

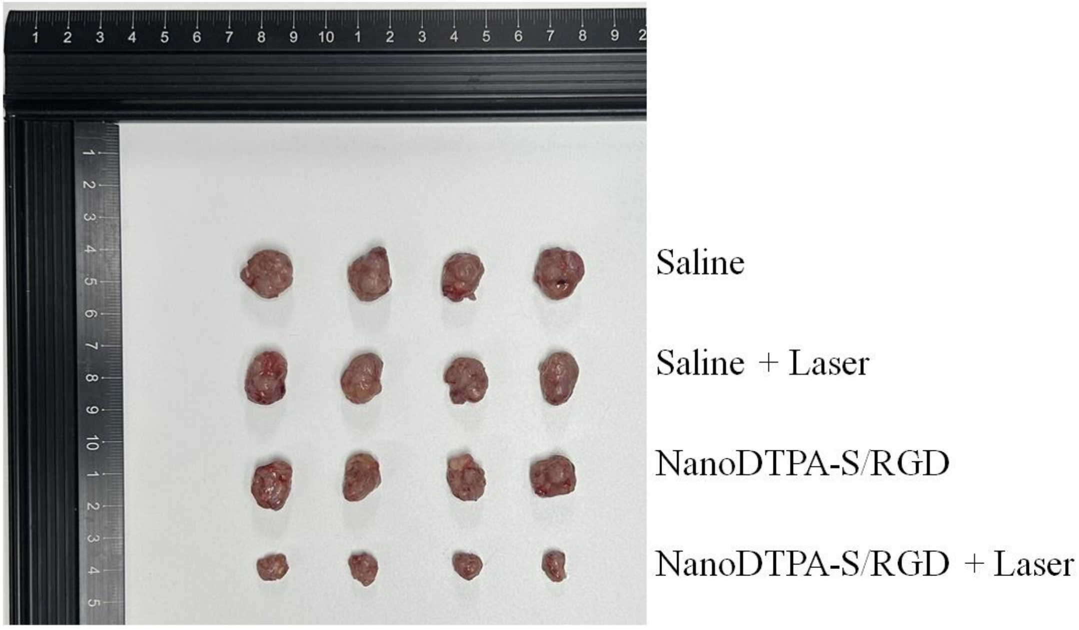

Figure S28. Tumor images from different groups of mice after 21 days' treatment.

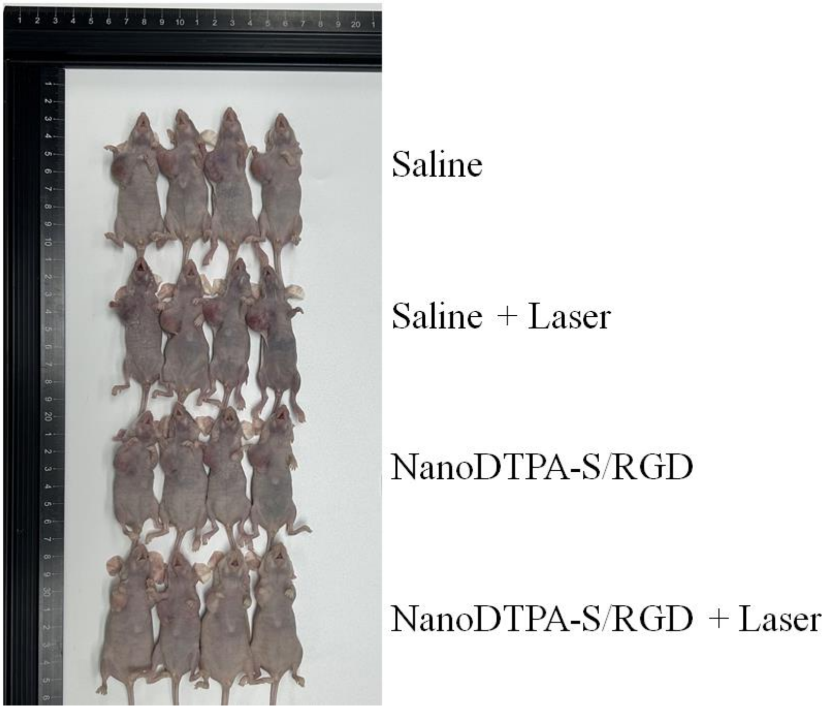

Figure S29. Photos of sacrificed mice from different groups after 21 days' treatment.

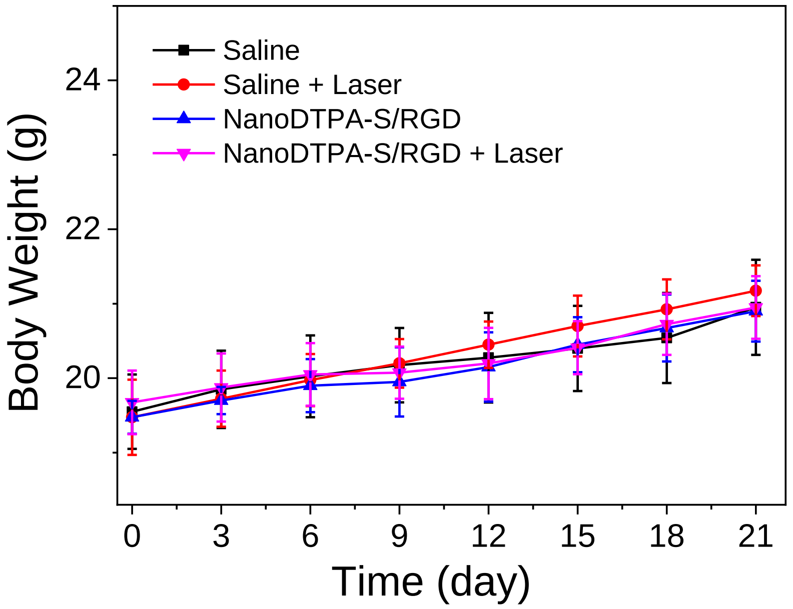

Figure S30. Body weight of different groups of mice.
